# Decomposing parasite fitness reveals the basis of specialization in a two‐host, two‐parasite system

**DOI:** 10.1002/evl3.65

**Published:** 2018-07-11

**Authors:** Eva J. P. Lievens, Julie Perreau, Philip Agnew, Yannis Michalakis, Thomas Lenormand

**Affiliations:** ^1^ UMR 5175 CEFE CNRS–Université de Montpellier–Université P. Valéry–EPHE 34293 Montpellier Cedex 5 France; ^2^ UMR 5290 MIVEGEC CNRS–IRD–Université de Montpellier 34394 Montpellier Cedex 5 France

**Keywords:** *Artemia*, ecological specialization, fecundity compensation, host specificity, microsporidians, multihost, multiparasite, parasite fitness, parasite life history, resistance

## Abstract

The ecological specialization of parasites–whether they can obtain high fitness on very few or very many different host species–is a determining feature of their ecology. In order to properly assess specialization, it is imperative to measure parasite fitness across host species; to understand its origins, fitness must be decomposed into the underlying traits. Despite the omnipresence of parasites with multiple hosts, very few studies assess and decompose their specialization in this way. To bridge this gap, we quantified the infectivity, virulence, and transmission rate of two parasites, the horizontally transmitted microsporidians *Anostracospora rigaudi* and *Enterocytospora artemiae*, in their natural hosts, the brine shrimp *Artemia parthenogenetica* and *Artemia franciscana*. Our results demonstrate that each parasite performs well on one of the two host species (*A. rigaudi* on *A. parthenogenetica*, and *E. artemiae* on *A. franciscana*), and poorly on the other. This partial specialization is driven by high infectivity and transmission rates in the preferred host, and is associated with maladaptive virulence and large costs of resistance in the other. Our study represents a rare empirical contribution to the study of parasite evolution in multihost systems, highlighting the negative effects of under‐ and overexploitation when adapting to multiple hosts.

**Impact summary**

Parasites have evolved many responses to the classic evolutionary dilemma “specialism versus generalism.” Some parasites are highly specialized, infecting only one host species, but many (maybe even most) parasites infect multiple hosts. Often, these multihost parasites are classified as relative specialists or generalists based on the number of hosts they infect, but this can lead to false conclusions about their evolution and epidemiology. Instead, assessing a parasite's degree of specialization should involve fitness measurements across its host range, and understanding the origin of that specialization requires a study of the individual fitness components: how do for example infectivity, virulence, and transmission vary across host species? Despite the ubiquity of multihost parasites and the acknowledged importance of decomposing their fitness across hosts, there are very few studies that take this essential step. In this study, we dissected the fitness of two microsporidian parasites, *Anostracospora rigaudi* and *Enterocytospora artemiae*, in their natural brine shrimp hosts *Artemia franciscana* and *Artemia parthenogenetica*. We show that each parasite performs much better on one host than on the other. More significantly, we discovered that the traits underlying this partial specialization are high infectivity and transmission rates in the preferred host. The tuning of virulence also plays an important role: in the nonpreferred hosts, the parasites manifest maladaptive virulence (overexploitation in one case, underexploitation in the other) and hosts incur large costs of resistance. These results, which highlight the difficulty of calibrating host exploitation across multiple host species, provide an important empirical contribution to our understanding of parasite evolution.

Life in a variable environment imposes an evolutionary choice between specializing to certain habitats and remaining a generalist. This dilemma is particularly pressing for parasitic species, which often come into contact with a wide range of potential habitats (i.e., hosts). Evolving an optimal level of specialization is not trivial, as adaptation to one host may come at the expense of adaptation to another (Levins 1968; Kawecki 1994; Kassen 2002). Furthermore, the degree of specialization affects the ecology and future evolution of the parasite: generalist parasites are more likely to survive perturbations in the host community and to colonize new hosts (Cleaveland et al. 2001; Agosta et al. 2010), while specialist parasites are more likely to interact tightly with their hosts (Kawecki 1998). The degree of specialization, therefore, is a key trait of parasite species. It varies widely among species–even within clades, parasites can range from extremely specific (infecting only one host species) to widely generalist (infecting tens of host species) (Poulin and Keeney 2008)–and through time–many parasites can evolve from generalism to specialism or vice versa when conditions change (e.g., Desdevises et al. 2002; Tanaka et al. 2007; Johnson et al. 2009; Cenzer 2016).

Thus, assessing how specialized multihost parasites are, and to which hosts, is an essential step to understanding and controlling the epidemiology and evolution of multihost parasites. To this end, the “ecological specialization” of parasites should be distinguished from the standard concepts “host range” and “host specificity” (sensu Lymbery 1989). Neither host range–the number of host species in which a parasite occurs–nor host specificity–host range weighted by infection intensity or host phylogeny–account for the existence of host species that barely contribute to the parasite's transmission. Such “spillover” hosts (sensu Fenton et al. 2015) can readily become infected, but do not transmit the parasite enough to keep its population growth rate above one. As a consequence, infection in the spillover hosts quickly dies out if there is no replenishing transmission from suitable hosts (“dead‐end” and “stuttering chain” dynamics, Viana et al. 2014). In essence, these are ecological source‐sink dynamics. Ecological specialization can take these dynamics into account: it is based on niche breadth (Futuyma and Moreno 1988), and sink habitats fall outside the fundamental niche (Pulliam 1988). Classifying organisms as ecological generalists or specialists means studying the variation in their fitness across a range of environments (Kassen 2002). Applied to parasites, this means their fitness must be assessed in all the affected host species. Such assessments typically require detailed epidemiological models (e.g., Rhodes et al. 1998; Fenton et al. 2015) or sizeable experiments (Jaenike and Dombeck 1998; Ahonen et al. 2006; Auld et al. 2017).

A second step is to understand why parasite fitness varies across hosts. The fitness of infections emerges from a suite of parasite‐ and host‐determined traits, including infectivity, exploitation of host resources, virulence, immune evasion, and transmission success. The nature of these traits has important consequences for a parasite: evolutionary constraints can emerge from functional correlations between traits within a host species (Walther and Ewald 2004; Alizon et al. 2009; Alizon and Michalakis 2015; Hall et al. 2017), or from correlations between the same trait in different host species (Futuyma and Moreno 1988; Via and Hawthorne 2002). They also determine the source of the parasite's maladaptation to spillover hosts (Woolhouse et al. 2001). This has been best studied with regards to virulence and transmission, mostly in single‐host systems (e.g., Dwyer et al. 1990; Fraser et al. 2007; de Roode et al. 2008; Doumayrou et al. 2012). Studies that decompose the fitness of multihost parasites into component traits are very rare (reviewed in Rigaud et al. 2010; see also Agudelo‐Romero et al. 2008; Auld et al. 2017).

Here, we examine specialization and its component traits in a natural multihost, multiparasite system. In the saltern of Aigues‐Mortes, France, two species of brine shrimp occur in sympatry: a native parthenogenetic clade, *Artemia parthenogenetica*, and an introduced sexual species, *Artemia franciscana* (Amat et al. 2005). Both *Artemia* species are parasitized by the microsporidians *Anostracospora rigaudi* and *Enterocytospora artemiae*. These microsporidians belong to a clade whose members mostly infect the intestinal epithelium of insects and crustaceans (Rode et al. 2013a). Accordingly, they have similar life cycles: they infect the gut epithelium, transmitting infection horizontally through spores released with the faeces (Rode et al. 2013a, b), and probably also through spores released from decaying hosts after death (Rode et al. 2013b; cf. Auld et al. 2017). Since spores are ingested through filter‐feeding and the host species are not spatially segregated at any given site (Lenz and Browne 1991), the pool of microsporidian spores is shared between *A. franciscana* and *A. parthenogenetica* (cf. Fels 2006). Although the rates of interspecific transmission should therefore be high, and both *A. rigaudi* and *E. artemiae* commonly infect either host species, the two microsporidians appear to be somewhat specialized: *A. rigaudi* is consistently more prevalent in *A. parthenogenetica*, while *E. artemiae* is more prevalent in *A. franciscana* (Rode et al. 2013a; Lievens et al. unpubl. data). Historically, the association of *A. parthenogenetica* and *A. rigaudi* predates the introduction of *A. franciscana* (in 1970, Rode et al. 2013c), while *A. franciscana* is also infected by *E. artemiae* in its native range (Rode et al. 2013c). It is not known whether *E. artemiae* was also present in France before the introduction of *A. franciscana*, whether it was cointroduced, or whether it arrived independently afterwards.

We evaluated parasite specialization in this system by studying the infectivity, virulence, and transmission of *A. rigaudi* and *E. artemiae* in each of their hosts. We confirm experimentally that while both microsporidians can complete their life cycle in the two host species, neither is a complete generalist. Rather, *A. rigaudi* is largely specialized on *A. parthenogenetica*, while *E. artemiae* is largely specialized on *A. franciscana*. Further, we show that the lower fitness of the two parasites in their nonspecialized hosts was caused by a reduction in infectivity and transmission rate (in both cases), combined with a suboptimal degree of virulence (too low for *E. artemiae*; too high for *A. rigaudi*). This demonstrates that a successful calibration of host exploitation and parasite virulence is central to the specialization of multihost parasites.

## Methods

We performed two experiments to investigate the life history and virulence of the microsporidians *A. rigaudi* and *E. artemiae* in their *Artemia* hosts. First, we used dose‐response tests to quantify infectivity in each host‐parasite combination. Second, we did a large‐scale experimental infection experiment, tracking individual host growth, mortality, and reproduction, as well as parasite transmission, over a period of two months. We provide an overview of the experimental procedures and statistical analyses below; more detail for each section can be found in the Supplementary Methods.

### EXPERIMENTAL CONDITIONS

The *Artemia* used in both experiments were raised in the lab in parasite‐free conditions. *A. franciscana* were hatched from dormant cysts sampled from the saltern of Aigues‐Mortes. We used three batches of cysts, sampled at the sites Caitive Nord or Caitive Sud in October 2013 or 2014. *A. parthenogenetica* were collected as live larvae from a mix of Aigues‐Mortes clones. Our stocks of *A. rigaudi* and *E. artemiae* contained a mix of spores from different Aigues‐Mortes sites and dates, collected from and propagated on both host species.

### SPORE COLLECTION AND QUANTIFICATION

To produce the inocula for our experiments, we collected fresh spores from the lab stocks of *A. rigaudi* and *E. artemiae*. Spore concentration was quantified using fluorescence microscopy.

### EXPERIMENT 1: INFECTIVITY

#### Experimental design and execution

Previously, we studied the infectivity of *A. rigaudi* and *E. artemiae* using single, uncontrolled spore doses (Rode et al. 2013a). Here, we quantified infectivity more precisely by exposing individual *A. parthenogenetica* and *A. franciscana* to a range of controlled spore doses and measuring the proportion of infected individuals. Hosts were exposed to 0, 400, 800, 1 600, 3 200, or 6 400 spores per individual; doses were replicated 20 times, except when spore availability was limiting (*E. rigaudi* on *A. parthenogenetica*: 16, 8, and 4 replicates for the doses 400, 3 200, and 6 400 spores per individual, respectively). Hosts were sacrificed after five days, and PCR‐tested for the presence of *A. rigaudi* or *E. artemiae*.

#### Statistical analyses

To analyze the dose‐response curves, we used four‐parameter log‐logistic modeling in R (package drc, Ritz and Strebig 2005; R Core Team 2014). Because we did not perform the *A. parthenogenetica* and *A. franciscana* experiments at the same time, we could not control for environmental effects. Thus, we simply tested if the dose‐response curves for *A. rigaudi* and *E. artemiae* were different within each host species using a likelihood ratio test. If the effect was significant, we went on to compare the parameters of the two resulting curves (“compParm” function in the drc package).

### EXPERIMENT 2: VIRULENCE AND TRANSMISSION

#### Experimental design and execution

To quantify the virulence and transmission rates of *A. rigaudi* and *E. artemiae*, we experimentally infected individual *Artemia* with controlled spore doses. Subadult *A. franciscana* males, *A. franciscana* females, and *A. parthenogenetica* females were divided into three treatments: “Controls,” “Exposure to *A. rigaudi*,” and “Exposure to *E. artemiae*,” which were replicated as permitted by spore and host availability (Table 1). *A. franciscana* hosts were subdivided into three blocks, determined by their cyst origin; *A. parthenogenetica* hosts were subdivided into two blocks, determined by the age of their batch. Hosts varied slightly in size, but size classes were evenly distributed across blocks and treatments. Spore doses were designed to be comparable while maximizing infection rate (see results of Experiment 1): 3 000 spores/individual for *A. rigaudi* and 2 500 spores/individual for *E. artemiae*. Because *A. parthenogenetica* had low infection rates with *E. artemiae*, a separate set of *A. parthenogenetica* was infected with 10, 000 *E. artemiae* spores per individual (Table 1).

**Table 1:** Number of replicates for the different treatments in Experiment 2

	Exposure to *A. rigaudi*		Exposure to *E. artemiae*				

Treatment:[spore dose]	[3 000 sp/i]		[2 500 sp/i]		[10, 000 sp/i]	Controls	
***A. franciscana***	**86 ♂**	**86 ♀**	**132 ♂**	**132 ♀**		**120 ♂**	**120 ♀**
Origin: Caitive Nord 2013	26 ♂	26 ♀	72 ♂	72 ♀		60 ♂	60 ♀
Origin: Caitive Nord 2014	30 ♂	30 ♀	30 ♂	30 ♀		30 ♂	30 ♀
Origin: Caitive Sud 2014	30 ♂	30 ♀	30 ♂	30 ♀		30 ♂	30 ♀
***A. parthenogenetica***		**96 ♀**		**96 ♀**	**33 ♀**		**96 ♀**
Batch: 34 ± 2 days old		48 ♀		48 ♀	18 ♀		48 ♀
Batch: 26 ± 2 days old		48 ♀		48 ♀	15 ♀		48 ♀

We then tracked each individual *Artemia* over a two‐month period. To measure the virulence of the parasites, we followed the host's life history: survival and reproductive output (for females) were recorded daily, and growth was measured on days 30 and 60. To estimate parasite fitness, we collected fecal samples at regular time points (on days 15, 30, 45, and 60) and used these to estimate the rate of spore production. For a subset of individuals, we also performed a transmission assay, which related spore production to host‐to‐host transmission, at two time points (days 30 and 60).

A key aspect of infection follow‐up experiments is knowing which individuals were infected after exposure to the parasite, and which were not. In our experiment, we could be sure of the infection status for almost all individuals that died on or after day 15 (the first spore collection date). Before that date, we could not exclude false negatives (see Supplementary Methods).

#### Statistical analyses: Virulence and transmission

We analyzed the results of this experiment in two major parts. First, we examined the virulence of infections (effect of the parasite on host survival, growth, reproduction, and overall fitness). In these analyses, we excluded all individuals that did not become infected after exposure to the parasite. We also excluded all individuals that died before day 15 (we could not be certain of infection status before this day, see above). To make sure that we were not missing important events occurring before this cutoff, we repeated all statistical models for exposed versus control individuals that died before day 15. Second, we analyzed parasite transmission (spore production rate, infectiousness, and overall fitness). Analyses were run in R version 3.4.2 (R Core Team 2014) using the packages lme4 (linear‐mixed models, Bates et al. 2015), survival (survival analyses, Therneau 2014), pscl (hurdle models, Zeileis et al. 2008), and multcomp (fuction “glht” for post‐hoc testing, Hothorn et al. 2008).

An overview of the analyses is given in Table 2; a detailed description can be found in the Supplementary Methods. Here, we only provide a brief description of our proxies for parasite fitness. For each infection, we used two measures of spore production as proxies. First, we calculated the ‘lifetime transmission success’: we summed the number of spores in the fecal samples taken on days 15, 30, 45, and 60 for each infection, then corrected this cumulative spore count by *p*, the average infectiousness of a single spore in a given host‐parasite combination (see Table 2). Second, we calculated an asymptotic growth rate from a standard Leslie matrix of the infection (see Supplemental methods), also corrected by the average infectiousness *p*. Both proxies implicitly account for host density and spore encounter rate, as the infectiousness *p* was calculated for the specific density of the transmission assay. While the lifetime transmission success is a measure of the basic reproduction number R_0_, which describes parasite fitness under stable endemic conditions, the asymptotic growth rate is a measure of the net population growth rate, which describes fitness under epidemic conditions (Frank 1996; Hethcote 2000); we included both measures because either situation can occur in the field. It should be noted that we calculate these proxies to compare parasite fitness under these specific standardized conditions. In the field, parasite fitness may differ due to for example the release of spores from dead hosts, spore sedimentation and spore death, variation in host demography (changes in age structure and density across seasons and basins) and variation in host “quality” (presence of other parasites, heterogeneous nutrition levels, different development time at different temperatures, etc.) (Alizon and Michalakis 2015).

**Table 2:** Overview of statistical analyses

Tested variable	Statistical models and tests	Fixed‐effect terms in the full model	Random/frailty terms
**Virulence of infections: *A. franciscana* and *A. parthenogenetica* analyzed separately**			
**Survival**	Survival models^1^ + LRT + Dunnett p.‐h.	Treatment^2^, Sex (*A. f*.), Size class, double interactions	Origin (*A. f*.), Batch (*A. p*.)
**Growth between days 1 and 30** 3	LMM + LRT + Dunnett p.‐h.	Treatment^2^ * Sex (*A. f*.)* Size class	Origin (*A. f*.), Batch (*A. p*.)
**Reproduction**			
Time until sexual maturity	Survival models^1^ + LRT + Dunnett p.‐h.	Treatment^2^ * Size class	Origin (*A. f*.), Batch (*A. p*.)
Probability of producing a clutch	Bernouilli GLMM + LRT + Dunnett p.‐h.	Treatment^2^ * Size class	Origin (*A. f*.), Batch (*A. p*.)
Rate of offspring production^4^^,^ 5 ^,^ a	LMM + LRT + Dunnett p.‐h.	Treatment^2^ * Size class	Origin (*A. f*.), Batch (*A. p*.)
Timing of offspring production^4^^,^ 6 ^,^ a	Neg. binomial GLMM + LRT + Dunnett p.‐h.	Treatment^2^ * (Elapsed % of reproductive period + Treatment^2^ * (Elapsed % of reproductive period^2^)	Individual, Origin (*A. f*.), Batch (*A. p*.)
Type of offspring produced^a^	Binomial GLMM + LRT + Dunnett p.‐h.	Treatment^2^ * Size class	Origin (*A. f*.), Batch (*A. p*.)
**Fitness (Lifetime reproductive success)** 4 ^,^ 7	Neg. binomial hurdle models + LRT + Dunnett p.‐h.	Treatment^2^ * Size class	NA
**Parasite transmission and fitness: infections of *A. franciscana* and *A. parthenogenetica* analyzed together**			
**Infectiousness of one spore, *p*** 8	LMM + LRT + Tukey p.‐h.	Recipient sp. * Parasite sp.	Individual
**Spore production rate** b			
Spore count^9^^,^ c	Neg. binomial GLMM + LRT + Tukey p.‐h.	Host sp. * Parasite sp.	Individual
Spore count ∼ dose^9^^,^ d	Neg. binomial GLMM + LRT	Dose	Individual
**Fitness** b			
Lifetime transmission success	Kruskal–Wallis tests + Dunn p.‐h.	Host‐parasite combination^2^	NA
Asymptotic growth rate	Kruskal–Wallis tests + Dunn p.‐h.	Host‐parasite combination^2^	NA

See Supplementary Methods for details.

^1^Survival models were parametric; the best survival distribution was chosen by AICc. ^2^ *A. parthenogenetica* exposed to low and high doses of *E. artemiae* treated separately. ^3^Most host growth occurred between days 1 and 30 (Table S2), so only this period was analyzed further. ^4^Offspring could be nauplii or cysts. These two offspring types were not directly comparable: they probably require different amounts of energy to produce, and we allowed mortality to occur before counting nauplii. To account for this, we repeated the tests with nauplii weighted twice, equally, or half as much as cysts, and based our conclusions on the overall pattern. ^5^Rate of offspring production  =  total number of offspring/length of the reproductive period. The length of the reproductive period was the difference between the date of death (or censoring) and the date of sexual maturity. ^6^Modeled as clutch size as a function of the elapsed proportion of the reproductive period. The reproductive period started at sexual maturity and ended at death (or censoring). ^7^LRS calculated as the total number of offspring produced over the study period. ^8^Calculated by fitting the results of the transmission assay to an independent action model with birth‐death processes. ^9^Spore count  =  the number of spores counted in the fecal sample; we did not transform the spore count to spores/mL (≈ spore count ^*^ 700) to avoid skewing the error distribution.

^a^Only for females that produced at least 1 clutch. ^b^Analyzed for infected individuals only. ^c^Excluded *A. p*. exposed to high doses of *E. artemiae*. ^d^Only for *A. p*. infected with *E. artemiae*.

GLMM, generalized linear‐mixed models; LMM, linear‐mixed models; LRT, likelihood ratio testing; P.‐h., post‐hoc tests; *A. f*., *A. franciscana*. *A. p*., *A. parthenogenetica*. sp., species.

^*^ Interactions between the factors were included. NA, not applicable.

#### Statistical analyses: Infection versus resistance

In most of the experimental host‐parasite combinations, a subset of exposed hosts did not become (detectably) infected. Hereafter, we refer to these individuals as resistant, because we found a posteriori differences in the proportion of such individuals across host‐parasite combinations, and in their life history traits compared to infected individuals and controls. As above, the analyses of these two aspects excluded all individuals who died before infection status could be definitively determined, that is those that died before day 15 of the experiment.

We analyzed the distribution of resistance across host‐parasite combinations using χ^2^ tests. There was substantial variation in infection outcome for the combinations *A. franciscana*‐*A. rigaudi*, and *A. parthenogenetica*‐*E. artemiae* (low dose) (see Results). To investigate whether there were costs of resistance in these combinations (Schmid‐Hempel 2003), we repeated the survival and reproduction analyses described above with an added *Resistant‐Infected‐Control* factor. We added or excluded this factor and its interactions with the other fixed effects, then compared all models using the corrected AIC. If the *Resistant‐Infected‐Control* factor was maintained in the best models, we used contrast manipulation and AICc‐based model comparison to detect how the three host categories (*Resistant*, *Infected*, *Control*) differed.

## Results

### EXPERIMENT 1: INFECTIVITY

**Figure 1:**
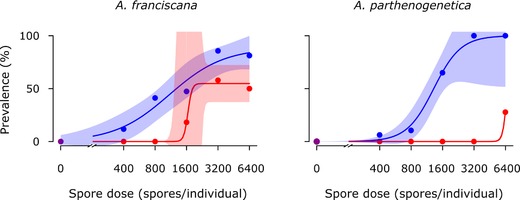
Infectivity of *A. rigaudi* (blue) and *E. artemiae* (red) in *A. franciscana* (left) and *A. parthenogenetica* (right). Points indicate the prevalence (% infected) at each dose; lines are the best fits and the shaded areas represent the 95% CIs. Because the inflection point of *E. artemiae* in *A. franciscana* was poorly resolved, uncertainty was high here. It was not possible to calculate a confidence interval for *E. artemiae* in *A. parthenogenetica* due to low resolution.

Both *A. parthenogenetica* and *A. franciscana* were more susceptible to infection with *A. rigaudi* than *E. artemiae* (χ^2^(3)  ≥ 20.9, *P*  <  0.001 for both; Fig. 1). For *A. franciscana*, the slopes and inflection points of the two curves were not significantly different, but the upper limit was significantly higher for *A. rigaudi* than for *E. artemiae* (*t*  =  2.1, *P*  =  0.03). In *A. parthenogenetica*, the infectivity of the two parasites was markedly different: successful infections with *E. artemiae* required such a high spore dose that the inflection point and upper limit of its curve could not be computed; its slope was not significantly different to that of *A. rigaudi*. Mortality was not dose‐dependent in any of the host‐microsporidian combinations, so we can be confident that it did not skew results (Table S1).

### EXPERIMENT 2: VIRULENCE AND TRANSMISSION

Among host individuals that survived until we could be certain of their infection status (i.e., that survived until at least day 15), infection rates were high (Table 3). As expected, many fewer infections were detected among individuals that died before day 15. In general, infection rates in Experiment 2 were considerably higher than those in Experiment 1; this was most likely because the longer incubation time allowed slow‐growing infections to become detectable.

**Table 3:** Detection of infection before and after the detection threshold (day 15)

Host‐parasite combination	Infection rate after vs. before the detection threshold
***A. franciscana***	
Exposure to *A. rigaudi*	86% vs. 50%
Exposure to *E. artemiae*	96% vs. 13%
***A. parthenogenetica***	
Exposure to *A. rigaudi*	100% vs. 15%
Exposure to *E. artemiae* – low spore dose	64% vs. 0%
Exposure to *E. artemiae* – high spore dose	86% vs. 20%

### VIRULENCE OF INFECTIONS

**Figure 2:**
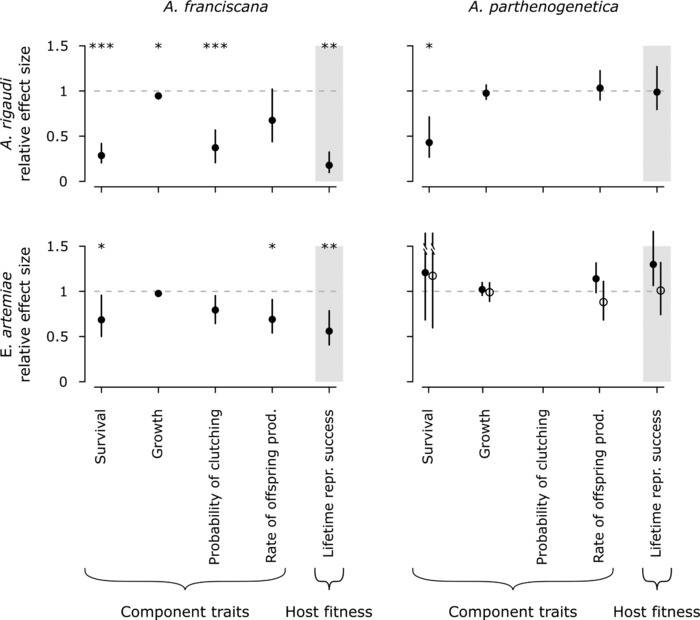
Host fitness (≈ parasite virulence) in the four host‐parasite combinations. All factors are shown as fitted effects relative to controls: survival is an acceleration factor (the ratio of expected time‐until‐death); the probability of reproduction is a relative risk; growth, rate of offspring production, and LRS are ratios. Bars represent the 95% profile likelihood CIs (survival) or bootstrapped CIs (all others). *A. parthenogenetica* infected after exposure to 10 ,000 *E. artemiae* spores are indicated with open circles. Asterisks indicate significant differences from controls (represented by the dotted gray line). The plotted survival effect for *A. parthenogenetica* excludes the aberrant group (see Results). All reproductive and fitness traits were obtained for females only. The probability of reproduction is not shown for *A. parthenogenetica* because it could not be analyzed. Weighing the contributions of nauplii and cysts to the rate of offspring production and LRS generated qualitatively equivalent results; the results shown here are for equal weights.

We analyzed the species‐level results of Experiment 2 in two parts. First, we analyzed the virulence of parasite infections, expressed as effects on host survival, growth, and reproduction. These results are summarized in Fig. 2 and the significance of tested effects is listed in Table 4; we discuss the effects of infection in more detail below. Here, we report only the analyses for infected versus control individuals, which excluded all individuals that died before day 15. When we compared exposed versus control individuals that died before the cut‐off day the results were not qualitatively different.

**Table 4:** Significance of tested effects for the virulence of infections

Tested variable	Fixed‐effect terms	Test statistic, *P*	Effect
**Virulence of infections: *A. franciscana***			
**Survival**	Treatment Sex Size class interactions	χ^2^(2) = 48.2, *P* < 0.0001 χ^2^(1) = 33.2, *P* < 0.0001 χ^2^(1) = 4.3, *P* = 0.04 all nonsignificant	↓ when infected ↑ for males ↑ for larger individuals
**Growth between days 1 and 30**	Treatment Sex Size class interactions	χ^2^(2) = 9.7, *P* < 0.01 χ^2^(1) = 133.5, *P* < 0.0001 χ^2^(1) = 95.0, *P* < 0.0001 all non‐significant	↓ for males ↓ for larger individuals
**Reproduction**			
Time until sexual maturity	Treatment Size class interaction	χ^2^(2) = 22.5, *P* < 0.0001 χ^2^(1) = 0.1, *P* = 0.83 χ^2^(2) = 1.3, *P* = 0.54	↑ when infected
Probability of producing a clutch	Treatment Size class interaction	χ^2^(2) = 31.3, *P* < 0.0001 χ^2^(1) = 0.5, *P* = 0.50 χ^2^(2) = 0.8, *P* = 0.69	↓ when infected
Rate of offspring production	Treatment Size class interaction	χ^2^(2) ≥ 7.9, *P* ≤ 0.02 † χ^2^(1) ≤ 0.5, *P* ≥ 0.50 † χ^2^(2) ≤ 1.8, *P* ≥ 0.41 †	↓ when infected
Timing of offspring production	Treatm.: % Repr. Period	χ^2^(4) ≤ 3.7, *P* ≥ 0.45†	
Type of offspring produced	Treatment Size class interaction	χ^2^(2) = 16.8, *P* < 0.001 χ^2^(1) = 0.1, *P* = 0.73 χ^2^(2) = 0.7, *P* = 0.71	more nauplii when infected
Fitness (LRS)	Treatment Size class Interaction	χ^2^(4) ≥ 46.6, *P* < 0.0001† χ^2^(2) ≤ 1.5, *P* ≥ 0.48† χ^2^(4) ≤ 1.5, *P* ≥ 0.82†	↓ when infected
**Virulence of infections: *A. parthenogenetica***			
**Survival**	Treatment Size class interaction	χ^2^(3) = 19.7, *P* < 0.001 χ^2^(2) = 11.5, *P* < 0.01 see text	↓ when infected ↑ for larger individuals
**Growth between days 1 & 30**	Treatment Size class interaction	χ^2^(3) = 1.3, *P* = 0.73 χ^2^(2) = 35.8, *P* < 0.0001 see text	↓ for larger individualssee text
**Reproduction**			
Rate of offspring production	Treatment Size class interaction	χ^2^(3) ≤ 5.8, *P* > 0.12† χ^2^(2) ≥ 8.8, *P* = 0.01† χ^2^(6) ≤ 10.4, *P* ≥ 0.11†	↑ for larger individuals
Timing of offspring production	Treatm.: % Repr. Period	χ^2^(4) ≥ 10.4, *P* < 0.11†	earlier when infected
Type of offspring produced	Treatment Size class interaction	χ^2^(2) = 1.4, *P* = 0.71 χ^2^(2) = 0.1, *P* = 0.96 χ^2^(6) = 9.1, *P* = 0.17	
**Fitness (LRS)**	Treatment Size class interaction	χ^2^(6) ≤ 5.6, *P* ≥ 0.13† χ^2^(4) ≥ 8.2, *P* < 0.09† χ^2^(12) ≤ 13.8, *P* ≥ 0.31†	↓ for largest individuals

^†^Depending on the weight of nauplii versus cysts.

Analyses were run separately for *A. franciscana* and *A. parthenogenetica*. See text for post‐hoc analyses of treatment.

In most host‐parasite combinations, survival was reduced (Fig. 2, Table 4). For *A. franciscana*, a lognormal survival model best fit the data (ΔAICc  ≥ 4.2). Infection significantly reduced survival; post‐hoc testing revealed that this effect was highly significant for *A. rigaudi* and marginally significant for *E. artemiae* (*t*  =  −6.7 and −2.2, *P*  <  0.0001 and *P*  =  0.05, respectively). For *A. parthenogenetica*, a log‐logistic survival model best fit the data (ΔAICc  ≥ 0.9). Survival was affected by infection, size class, and their interaction, but this complicated interaction effect was due to the aberrant survival curves of one group of individuals (Batch 34 ± 2 days old, Size class 7.5 mm), which had high death rates for controls and low death rates for infected hosts. When this group was removed, the interaction effect became nonsignificant. In general therefore, survival of *A. parthenogenetica* was reduced by infection with a parasite; post‐hoc testing revealed that individuals infected with *A. rigaudi* had significantly lower survival (*t*  =  –3.3, *P*  <  0.01), while individuals infected with *E. artemiae* did not (*t*  =  0.7 and 0.5, *P*  =  0.86 and 0.94 for low and high spore dose, respectively).

More than 90% of host growth occurred between days 1 and 30 (Table S2), so only this period was analyzed (Fig. 2, Table 4). For *A. franciscana*, infection significantly reduced growth; this effect was driven by *A. rigaudi* (post‐hoc *z*  =  −3.1, *P*  <  0.01) and nonsignificant for *E. artemiae* (post‐hoc *z*  =  −1.5, *P*  =  0.23). *A. parthenogenetica* growth was affected by infection interacting with size class, but this interaction produced incorrect predictions and a poor model fit. When it was removed, there was no effect of infection on growth.

Parasite infection affected the reproduction of *A. franciscana* females in various ways (Fig. 2, Table 4). The time until maturity, which was best described by a lognormal distribution (ΔAICc  ≥ 5.1), was significantly delayed by infection with either parasite species (post‐hoc for *A. rigaudi t*  =  4.6, *P*  <  0.0001; post‐hoc for *E. artemiae t*  =  2.5, *P*  =  0.02). The probability that *A. franciscana* females produced a clutch was also significantly lower when they were infected; this effect was driven by *A. rigaudi* (post‐hoc *z*  =  −5.3, *P*  <  0.0001) and was marginally nonsignificant for *E. artemiae* (post‐hoc *z*  =  –2.1, *P*  =  0.06). For *A. franciscana* females that did reproduce, infection with *A. rigaudi* increased the proportion of nauplii clutches (post‐hoc for *A. rigaudi z*  =  3.9, *P*  <  0.001; post‐hoc for *E. artemiae z*  =  0.8, *P*  =  0.68). The rate of offspring production was significantly reduced by infection with *E. artemiae* for all weights of nauplii versus cysts (post‐hoc for *E. artemiae z*  ≤  –2.8, *P*  ≤  0.01), but was only significantly reduced by infection with *A. rigaudi* when cysts were weighted twice as much as nauplii (*z*  =  −2.3, *P*  =  0.04). Finally, the timing of offspring production was independent of infection status.

**Figure 3:**
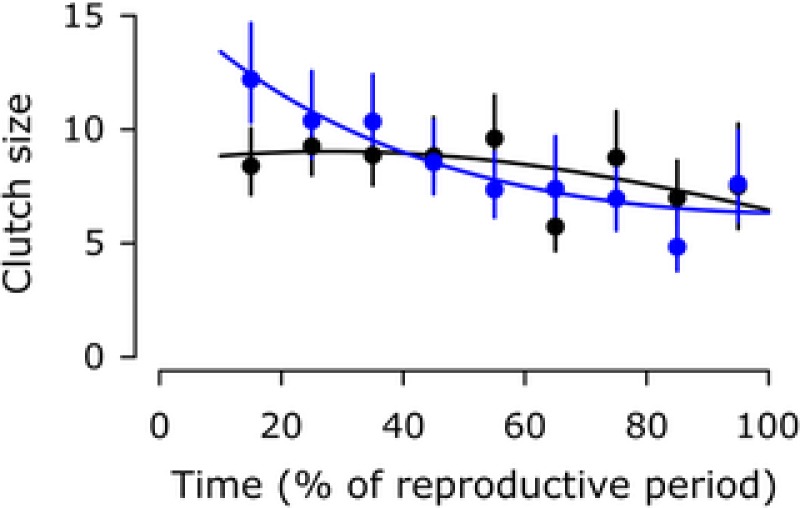
Timing of reproduction in *A. parthenogenetica* controls (black) and infected with *A. rigaudi* (blue). Lines represent the prediction of the best model, points and vertical bars give the observed means and their 95% CIs, calculated over intervals of 10%. Weighing the contributions of nauplii and cysts to the total number of offspring generated qualitatively similar results; the results shown here are for equal weights.

In contrast, parasite infection had little effect on the reproduction of *A. parthenogenetica* females (Fig. 2, Table 4). The effects of infection on the time until sexual maturity or the probability of producing a clutch could not be tested, because almost all *A. parthenogenetica* females started reproducing immediately. For reproducing females, neither the proportion of live clutches, nor the rates of offspring production were affected by infection with either parasite. However, infection with *A. rigaudi* did lead to a significant shift toward earlier reproduction (Fig. 3; significant effect of treatment when cysts were weighted equally or doubly compared to nauplii; post‐hoc for *A. rigaudi z*  ≥ 2.6, *P*  ≤  0.03 for all weights of nauplii vs. cysts; post‐hoc for *E. artemiae z*  ≤  1.1, *P*  ≥ 0.58 for all weights of nauplii vs. cysts).

The fitness of female hosts–estimated by the lifetime reproductive success (LRS), that is the total number of offspring produced–was significantly reduced by infection with either parasite for *A. franciscana* (post‐hoc for *A. rigaudi t*  ≤  −7.3, *P*  ≤  0.0001 for all weights of nauplii vs. cysts; post‐hoc for *E. artemiae t*  ≤  −3.9, *P*  ≤  0.001 for all weights of nauplii vs. cysts), but not for *A. parthenogenetica* (Fig. 2, Table 4).

#### Transmission and fitness of infections

**Figure 4:**
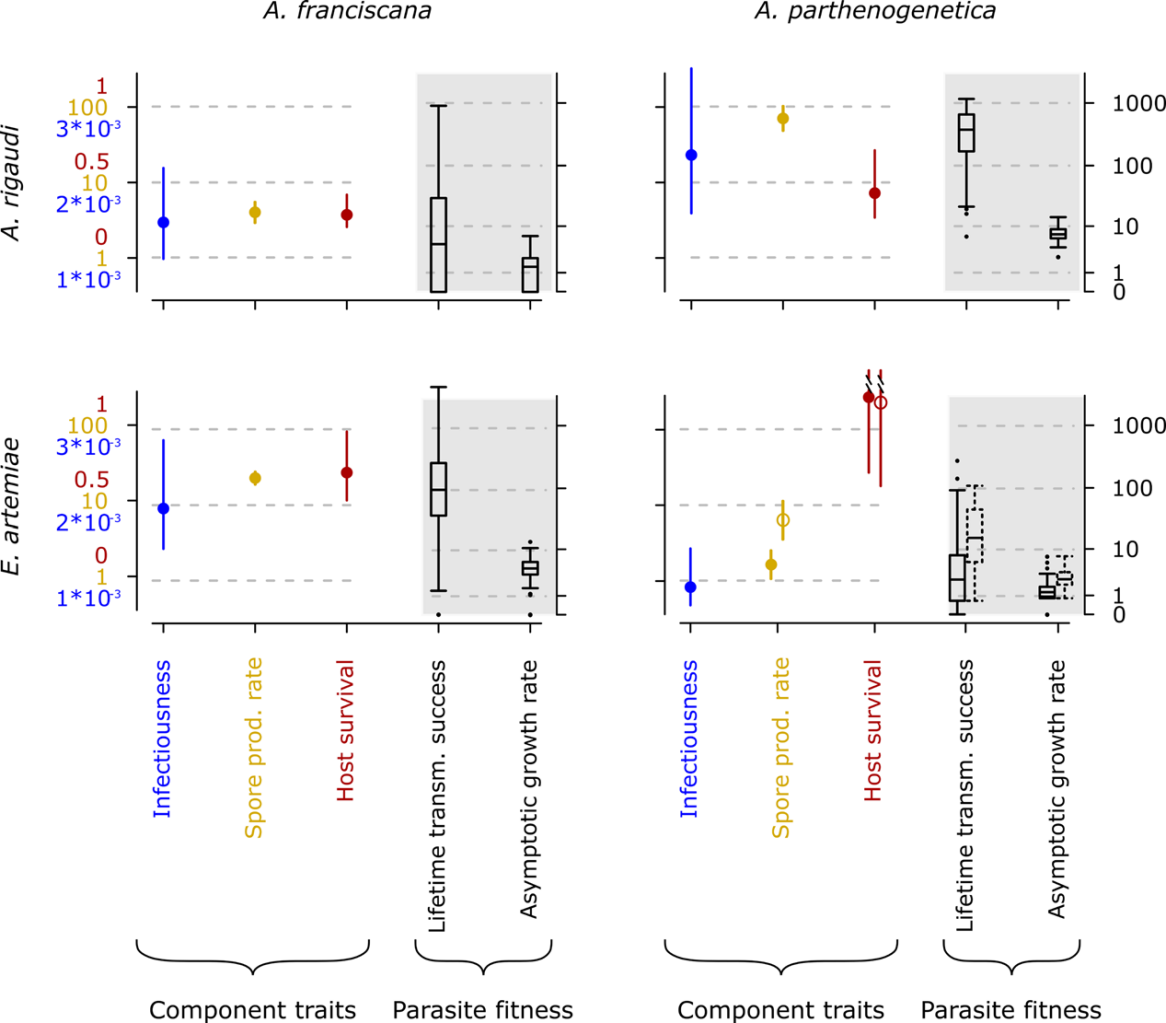
Parasite fitness in the four host‐parasite combinations. The component traits infectiousness (probability of infection by a single spore), rate of spore production (# counted spores/5 days, *ln* scale), and host survival (which determines infection duration, copied from Fig. 2) are shown as fitted means with 95% profile likelihood CIs. The fitness measures lifetime transmission success (*ln + 1* scale) and asymptotic growth rate (*ln + 1* scale) are shown as Tukey box plots. *A. parthenogenetica* infected after exposure to 10, 000 *E. artemiae* spores are indicated with open circles and dotted box plots. Note that spore production, host survival, and parasite fitness were analyzed for infected hosts only.

Second, we studied the effects of the host species on the parasite's transmission and fitness (summarized in Fig. 4). These analyses were combined for all host‐parasite combinations, but *A. parthenogenetica* that were exposed to 10, 000 *E. artemiae* spores were analyzed separately unless otherwise specified.

The infectiousness of a single spore (the probability that it started a detectable infection, as calculated using the transmission data) corresponded with our expectations based on Experiment 1 (Fig. 4). Host‐parasite combination had a significant effect on infectiousness (χ^2^(1)  =  16.7, *P*  <  0.0001). *A. rigaudi* tended to be more infectious to *A. parthenogenetica* than to *A. franciscana* (post‐hoc *z*  =  2.3, *P*  =  0.10); *E. artemiae* was significantly more infectious to *A. franciscana* than to *A. parthenogenetica* (post‐hoc *z*  =  3.6, *P*  <  0.01).

The rates of spore production were significantly different in all host‐parasite combinations (overall χ^2^(1)  =  205.9, *P*  <  0.0001; all post‐hoc pairwise comparisons *z*  ≥ 3.2, *P*  <  0.01; Fig. 4); they were highest in the combinations *A. parthenogenetica‐A. rigaudi* and *A. franciscana‐E. artemiae*. For *A. parthenogenetica* infected with *E. artemiae*, the rate of spore production was notably higher when the initial inoculum was larger (χ^2^(1)  =  10.6, *P*  =  0.001).

As expected, host‐to‐host transmission success increased with the rate of spore production in all host‐parasite combinations (Spearman's *ρ* between 0.57 and 0.69, *P*  <  0.0001; Fig. S1). Therefore, we were able to use the lifetime transmission success and asymptotic growth rate as indicators of parasite fitness. The two measures were tightly correlated (Fig. S2) and both differed across host‐parasite combinations (χ^2^(4)  =  189.9 and 245.0, respectively, *P*  <  0.0001; Fig. 4). The fitness of *A. rigaudi* infections was highest in *A. parthenogenetica*; that of *E. artemiae* infections was highest in *A. franciscana*. All pairs of host‐parasite combinations were significantly different, except the low performers *A. parthenogenetica‐E. artemiae* and *A. franciscana‐A. rigaudi* (post‐hoc *P*  <  0.001 vs. *P*  =  0.46 and *P*  ≤  0.04 vs. *P*  =  0.69, respectively). This was true for both spore doses of *A. parthenogenetica‐E. artemiae* regarding the lifetime transmission success, but only for the low spore dose for the asymptotic growth rate.

#### Infection versus resistance

Among the individuals that survived until we could be certain of their infection status, the rate of resistance varied between 0 and 36% in the different host‐parasite combinations (Table 3). For *A. franciscana*, significantly more individuals resisted infection with *A. rigaudi* than with *E. artemiae* (14% vs. 4%, χ^2^(1)  =  10.3, *P*  <  0.01), and this effect was independent of sex (χ^2^(1)  =  0.3, *P*  =  0.60). Inversely, significantly more *A. parthenogenetica* resisted infection with *E. artemiae* than with *A. rigaudi* (≥ 14% vs. 0%, χ^2^(1)  =  20. 6, *P*  <  0.0001), with a marginally non‐significant difference between the two spore doses (low dose 36% vs. high dose 14%, χ^2^(1)  =  3.8, *P*  =  0.052). There was substantial variation in infection outcome for the combinations *A. franciscana*‐*A. rigaudi*, and *A. parthenogenetica*‐*E. artemiae* (low dose), so we continued our analyses with these combinations.

**Figure 5:**
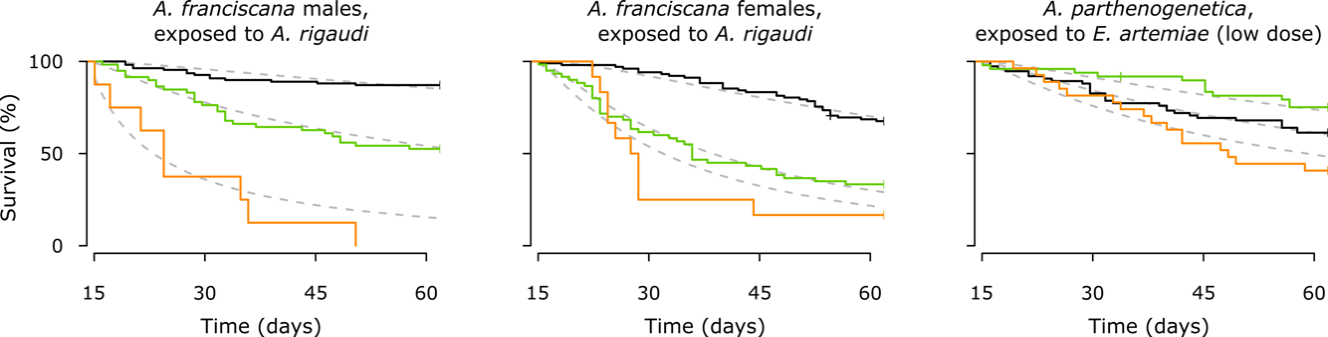
Survival curves for resistant (orange), infected (green), and control (black) individuals. Note that these curves start at day 15, that is when infection status could be fully ascertained. The curves shown here are averaged across size class and origin for *A. franciscana* and across size classes in *A. parthenogenetica*. Model estimates for each curve are plotted in gray.

For both *A. franciscana* exposed to *A. rigaudi* and *A. parthenogenetica* exposed to a low spore dose of *E. artemiae*, resistant individuals died more quickly than infected individuals (Fig. 5; ΔAICc, respectively > 4.4 and  =  1.6, Table S3). For *A. franciscana*, resistant males had a higher mortality than resistant females (Fig. 5; ΔAICc > 1.7, Table S3).

Finally, there was little support for an effect of resistance on reproduction in females of either host species (Table S4). *A. franciscana* females that resisted infection with *A. rigaudi* behaved similarly to females that became infected (strong effects of *Resistant‐Infected‐Control*, but no or weak support for a difference between resistant and infected females). The reproductive behavior of *A. parthenogenetica* females that resisted infection with a low dose of *E. artemiae* was similar to that of infected and control females.

## Discussion

The degree of host specialization is a key property of any multihost parasite. Host specialization, when considered as a difference in fitness, arises from a series of life history traits including the ability to infect, the rate of transmission, and the virulence. We quantified these traits for two microsporidian gut parasites (*A. rigaudi* and *E. artemiae*) infecting two brine shrimp hosts (*A. franciscana* and *A. parthenogenetica*), by tracking the life history of both hosts and parasites after experimental infection. A brief synopsis of the results is shown in Table 5.

**Table 5:** Qualitative synopsis of results

	Parasite species	
Host species	*A. rigaudi*	*E. artemiae*
*A. franciscana*	Moderately infectious Low spore production Highly virulent **⇨Low parasite fitness**, **mismatched host & parasite**	Highly infectious High spore production Moderately virulent **⇨High parasite fitness**, **matched host & parasite**
*A. parthenogenetica*	Highly infectious High spore production Moderately virulent (survival only) **⇨High parasite fitness**, **matched host & parasite**	Poorly infectious Low spore production Avirulent **⇨Low parasite fitness**, **mismatched host & parasite**

Overall, each of the parasites was partially specialized: *A. rigaudi* was very successful in *A. parthenogenetica*, while *E. artemiae* performed best in *A. franciscana*. Below, we discuss how the individual life history traits combine to shape the degree of specialization, and the ensuing effects of specialization on the hosts. We refer to the host‐parasite combinations where parasites reached high fitness as the “matched” combinations (Table 5). The reversed combinations also produced viable transmission stages, but at much lower rates; we will call these the “mismatched” combinations.

### PARTIAL SPECIALIZATION VIA A MIX OF SPECIALIST AND GENERALIST TRAITS

Specialization is often presented as a dichotomy: specialists, whose fitness is high or null for different hosts, versus generalists, who generally have intermediate fitness on several hosts (Poulin 2007; Schmid‐Hempel 2011; Leggett et al. 2013). *A. rigaudi* and *E. artemiae* fall into a gray zone between these categories, being neither absolute specialists–they can exploit both hosts–, nor absolute generalists–their fitness is much higher in the matched hosts.

When broken down into its component traits, the origin of this partial specialization becomes clear. Parasites should be as infective as possible to hosts to which they are adapted, and indeed both *A. rigaudi* and *E. artemiae* are highly infectious to their matched hosts. Similarly, we expect strong transmission to be advantageous, and accordingly we find that both parasites have high rates of spore production in their matched hosts. The expectations for virulence are not as clear‐cut. A “Darwinian devil” parasite would be avirulent while maintaining high transmission rates, but it is generally considered that these two factors are correlated (Alizon et al. 2009). Virulence must therefore be judged in relation to transmission; for example, high virulence can be adaptive if coupled with high rates of transmission, or maladaptive if not. When considered in this way, *A. rigaudi* and *E. artemiae*’s virulence are also coherent with their overall specialization. *A. rigaudi* causes high survival virulence–and thus short infection durations–in both hosts, but this is advantageously coupled with high rates of spore production in its matched host *A. parthenogenetica*, and disadvantageously coupled with low rates of spore production in its mismatched host. *E. artemiae* is avirulent in its mismatched host, which at first glance appears ideal. However, when spore production is taken into account, it becomes clear that this avirulence in *A. parthenogenetica* is coupled with very low rates of transmission, whereas the rate of spore production is high in *A. franciscana*.

Despite this, were we to consider the component traits individually, they would not all lead us to conclude that the two parasites are partially specialized. The pattern of spore production in the matched versus mismatched combinations best reflects the overall degree of specialization. Infectivity, on its own, might lead us to conclude that *E. artemiae* is a specialist while *A. rigaudi* is more generalist. Virulence is difficult to interpret outside the context of spore production, as discussed above, making it a particularly poor proxy for overall specialization. Integrating across all of these life history traits is therefore necessary to properly understand the nature of this host‐parasite system, and will probably have important implications for the evolution of virulence (Alizon and Michalakis 2015) and infection success (Hall et al. 2017).

### MISMATCHED PARASITES HAVE DIFFERENT KINDS OF SUBOPTIMAL VIRULENCE

Several theoretical predictions have been made for the evolution of virulence in multihost parasites that are specialized on one host and spill over into another (source‐sink dynamics), all of which agree that virulence should depend exclusively on the optimum in the specialized host (Regoes et al. 2000; Woolhouse et al. 2001; Dobson 2004; Gandon 2004). Predictions of virulence in the nonspecialized host, however, vary. Regoes et al. considered virulence to be coupled to exploitation, which trades off between hosts; their prediction is that the parasite will be avirulent in the spillover host. Gandon also considered virulence to be coupled to exploitation, but in his model the level of exploitation is correlated between hosts. In this case, the parasite can be maladaptively avirulent or hypervirulent in the spillover host, depending on the relative resistances of the hosts. Finally, Woolhouse et al. pointed out that virulence can become decoupled from parasite exploitation in spillover hosts, for example through harmful immune responses (Graham et al. 2005), leading to maladaptively high virulence (see also Leggett et al. 2013). Empirically, virulence patterns across multiple hosts have only rarely been studied in natural systems (Rigaud et al. 2010), so it is difficult to determine which of these possibilities may be more common.

In the mismatched hosts of our *Artemia*‐microsporidian system, two different virulence patterns are apparent. First, in the combination *A. franciscana‐A. rigaudi*, the parasite is very virulent on a host in which it can barely reproduce. Its virulence in the nonspecialized host is thus decoupled from exploitation and maladaptive, matching Woolhouse et al.’s (2001) prediction for unconstrainedly high virulence. The situation of *A. rigaudi* strongly resembles that of the generalist microsporidian parasite *Nosema bombi*, which infects bumble bees (Rutrecht and Brown 2009); a number of zoonotic human diseases also fit this pattern (Woolhouse et al. 2001; cf. Auld et al. 2017). In contrast, in the mismatched combination *A. parthenogenetica‐E. artemiae*, the parasite is avirulent. *E. artemiae* could therefore correspond to the situations described by Regoes et al. (2000) and Gandon (2004), in which a nonspecialized host is underexploited and suffers no virulence. Indeed, *A. parthenogenetica* is also less susceptible to *E. artemiae*, giving some support to Gandon's scenario of differently resistant hosts. A similar case could be made for the nematode *Howardula aoronymphium* (Jaenike 1996; Jaenike and Dombeck 1998; Perlman and Jaenike 2003) and for the Drosophila C virus (Longdon et al. 2015), which exhibit a range of exploitation and correlated virulence across host species.

Overall, our results provide support for the varied possible theoretical predictions of virulence evolution in multihost parasites: in one case, we appear to be dealing with decoupled, “runaway” virulence, while in the second the differences in virulence may be driven by levels of host resistance. These contrasting findings show that the different theoretical outcomes can even be found among host‐parasite pairs that are ecologically extremely similar and phylogenetically close.

### MISMATCHED HOSTS INCUR HIGH COSTS OF RESISTANCE

In the matched host‐parasite combinations, uninfected individuals were rare or nonexistent (Table 3), and suffered no detectable survival cost (data not shown). It is possible that an extremely high mortality rate of resistant individuals caused them to die before we could reliably detect infection, leading us to underestimate both the frequency and the cost of resistance. However, survival rates for the matched combinations were universally high in the infectivity experiment, which lasted one week. Any mortality conferred by resistance would therefore have to be incurred precisely in the second week of infection, which is unlikely. It is more probable that the high rates of infection reflect selection on the parasite to evade or overcome resistance in its matched host (Hasu et al. 2009).

In the mismatched host‐parasite combinations, however, up to one third of the exposed hosts were uninfected, and the life histories of these individuals differed clearly from those of control or infected hosts (Table 3, Fig. 5). This suggests that their lack of infection was the result of an active resistance mechanism. Because the parasite was absent, the effects of deploying resistance must have been induced by the host itself, as a consequence of its immune reaction upon exposure (immunopathology, Schmid‐Hempel 2003; Graham et al. 2005).

This resistance was extremely costly: resistant individuals died much more rapidly than control and infected hosts (Fig. 5). Since there was no detectable compensation through increased fecundity, we must conclude that resistance in these cases is maladaptive. This is intriguing, because *A. franciscana* and *A. parthenogenetica* are regularly exposed to their mismatched parasites in the field (Rode et al. 2013c). Host resistance has been shown to evolve quickly in a similar host‐parasite system (*Daphnia magna‐Octosporea bayeri*, Zbinden et al. 2008), so we would not expect maladaptive resistance responses to persist in the host populations. An explanation may be that source‐sink dynamics acting in the parasite populations prevent them from evolving to reduce their impact on the mismatched hosts. In turn, selection on the host to reduce its response to the mismatched parasite could perhaps be countered by other factors, such as the need to maintain its overall immune capacity (Graham et al. 2005). Similarly disproportionate costs of resistance, with uninfected hosts dying more rapidly than even infected hosts, have been found in for example *Daphnia* resisting the bacterium *Pasteuria* (Little and Killick 2007; though see Labbé et al. 2010), and naïve isopods resisting infection with a helminth (Hasu et al. 2009).

### INFECTION WITH *A. RIGAUDI* CAUSES SHIFTS IN REPRODUCTIVE STRATEGY

*A. parthenogenetica* females infected with their matched parasite *A. rigaudi* died more quickly than controls and did not produce offspring at a higher overall rate, yet did not suffer from reduced lifetime reproductive success. They managed this by shifting toward earlier reproduction to alleviate the survival virulence, a plastic behavior known as fecundity compensation (cf. Minchella and Loverde 1981; Agnew et al. 2000; Chadwick and Little 2005) (Fig. 3). Females accomplished this shift in reproductive effort by increasing the size, rather than the frequency, of early clutches (frequency data not shown). This is a new finding for *Artemia*, which could cast a new light on the relationship between Mediterranean *A. parthenogenetica* and their castrating cestode parasite *Flamingolepis liguloides* (Amat et al. 1991).

*A. franciscana* females did not have a similar fecundity compensation response when infected with either parasite. However, infections of *A. franciscana* with *A. rigaudi* were associated with an interesting change in reproductive strategy. Infected females were less likely to produce a clutch, but those that did reproduce were more likely to produce clutches of live young. Considering that *Artemia* generally produce cysts when stressed (Clegg and Trotman 2002), this result seems counterintuitive. Perhaps *A. rigaudi* interferes with the cyst production mechanism, either collaterally or as a manipulation to increase the availability of susceptible hosts. Another possibility is that a shift toward live born offspring is advantageous for the host. If infected mothers can produce offspring that are protected against the parasite, for example via transgenerational immune priming (which Artemia can do, Norouzitallab et al. 2015), those offspring should have a competitive advantage when encountering the parasite. If this protection is costly, it may be more worthwhile to produce protected nauplii than protected cysts: protected nauplii will certainly be born into a parasite‐infested environment, while the hatching environment of protected cysts is unknown.

### COMPARISON WITH THE FIELD: PREVIOUS AND FUTURE RESULTS

Quite remarkably, the results of this study are consistent with all the field observations and previous laboratory results of the *Artemia*‐microsporidian system. Our identification of the matched and mismatched host‐parasite combinations reflects the consistently higher prevalence of *A. rigaudi* and *E. artemiae* in respectively *A. parthenogenetica* and *A. franciscana* (Rode et al. 2013c; Lievens et al. unpubl. data). In addition, we find that *A. rigaudi* is considerably more virulent than *E. artemiae* in both host species. Rode et al. (2013c) reached a similar conclusion based on the reproductive state of females collected from the field. Interestingly, the effect found by Rode et al. was that sexually mature females of both species were less likely to be brooding a clutch when they were infected with *A. rigaudi*, while in our study *A. rigaudi* did not affect the frequency of clutching once sexual maturity had been reached (data not shown). The different conditions in the field may be responsible for this seemingly additional virulence (e.g., food limitation, Brown et al. 2000, Bedhomme et al. 2004, Vale et al. 2011; temperature, Mitchell et al. 2005, Vale et al. 2008). In the future, it would be interesting to extend our comparison of field and lab results to parasite fitness. In particular, our experimental conditions should allow parasite persistence in all the host‐parasite combinations (median parasite fitness above one, Fig. 4). However, natural conditions are less generous (e.g., lower host density, higher host mortality, higher risk of spore death; Alizon and Michalakis 2015), so parasite fitness in the field is probably lower overall–it may be that the persistence of *A. rigaudi* and *E. artemiae* requires certain host combinations (Fenton et al. 2015).

Having established that the parasites are partially specialized, we can ask to what extent this situation maximizes parasite fitness in the field. For example, currently *A. parthenogenetica* is a higher quality host for *A. rigaudi* than *A. franciscana*. Nonetheless, in conditions where *A. parthenogenetica* is rare, it could be advantageous for *A. rigaudi* to evolve away from specialization on the high‐quality host and toward the exploitation of the more numerous poor‐quality host. The answer to this question depends on the relative quality and quantity of the two host species (Kassen 2002), and as such cannot be answered by our lab‐based fitness measures (see above). Instead, answers could come from tracking the evolution of the parasite populations in nature (cf. Tanaka et al. 2007; Cenzer 2016) or in experimental evolutions.

Further insights into the relationship between the microsporidians and their *Artemia* hosts could come from experimental coinfections. So far, we have examined the effects of *A. rigaudi* and *E. artemiae* in isolation, but coinfections are very common in the field (Lievens et al. unpubl. data). Coinfection often has profound effects on the expression of parasite virulence and the success of their transmission, and can thus be expected to affect the evolution of microsporidian life history and host responses (Rigaud et al. 2010; Alizon et al. 2013). Studying the effects of single versus mixed infections could therefore provide new perspectives into selection on ecological specialization in the field.

### CONCLUSION

In nature, multihost parasites and multiparasite hosts are likely to be the rule, rather than the exception (Cleaveland et al. 2001; Taylor et al. 2001; Streicker et al. 2013). Despite important research efforts in these complex systems, we still know little about the interplay between parasite specialization and its component traits (Rutrecht and Brown 2009; Rigaud et al. 2010; Hall et al. 2017). In this study, we dissected the fitness traits involved in parasite adaptation in all combinations of a naturally occurring two‐host, two‐parasite system. We showed that both parasites are partially specialized, with each performing better on one of the two host species. Furthermore, studying the underlying life history traits revealed that the heart of this specialization is the delicate balance between over‐ and underexploitation of the host: the drivers of infection success were spore production and the “tuning” of parasite virulence to match it. This occurred despite the ecological and phylogenetic similarity of the hosts and parasites, highlighting the difficulty of adapting (or not) to multiple host species.

## DATA ACCESSIBILITY

Data and analyses will be uploaded to Dryad upon acceptance.

Associate Editor: Prof. J. Slate

## Supporting information


**Table S1**. Host survival during the infectivity experiment.
**Table S2**. Results of paired t‐tests comparing host growth before and after day 30 (all treatments combined).
**Table S3**. Model comparison: link between survival and infection success.
**Table S4**. Model comparison: link between reproduction and infection success.
**Figure S1**. Spore production and host‐to‐host transmission success in the four host‐parasite combinations.
**Figure S2**. Overall fitness measures of *A. rigaudi* (top) and *E. artemiae* (bottom) infections.
Supplementary Methods
