# Supplementary material for: Decomposing parasite fitness reveals the basis of specialization in a two‐host, two‐parasite system
Source: Evol Lett. 2018 Jul 11;2(4):390–405. doi: 10.1002/evl3.65 (PMC6121826; doi:10.1002/evl3.65)
Supplement: Supplementary file 1 — Table S1. Host survival during the infectivity experiment. Table S2. Results of paired t‐tests comparing host growth before and after day 30 (all treatments combined). Table S3. Model comparison: link between survival and infection success. Table S4. Model comparison: link between reproduction and infection success. Figure S1. Spore production and host‐to‐host transmission success in the four host‐parasite combinations. Figure S2. Overall fitness measures of A. rigaudi (top) and E. artemiae (bottom) infections. Supplementary Methods [file EVL3-2-390-s001.docx]

## Supplementary tables & figures

**Table S1.** Host survival during the infectivity experiment.

| **Parasite species** | **Dose (spores/individual)** | **Nb. Exposed** | **Nb. Died** | **% Survived** |
| --- | --- | --- | --- | --- |
| ***A. franciscana*** |  |  |  |  |
| Controls | 0 | 17 | 7 | 59% |
| *A. rigaudi* | 400 | 20 | 3 | 85% |
|  | 800 | 20 | 3 | 85% |
|  | 1 600 | 20 | 1 | 95% |
|  | 3 200 | 20 | 6 | 70% |
|  | 6 400 | 20 | 4 | 80% |
| *E. artemiae* | 400 | 20 | 10 | 50% |
|  | 800 | 20 | 4 | 80% |
|  | 1 600 | 20 | 9 | 55% |
|  | 3 200 | 20 | 1 | 95% |
|  | 6 400 | 20 | 8 | 60% |
| ***A. parthenogenetica*** |  |  |  |  |
| Controls | 0 | 4 | 0 | 100% |
| *A. rigaudi* | 400 | 16 | 0 | 100% |
|  | 800 | 20 | 1 | 95% |
|  | 1 600 | 20 | 0 | 100% |
|  | 3 200 | 8 | 0 | 100% |
|  | 6 400 | 4 | 0 | 100% |
| *E. artemiae* | 400 | 20 | 2 | 90% |
|  | 800 | 20 | 0 | 100% |
|  | 1 600 | 20 | 1 | 95% |
|  | 3 200 | 20 | 2 | 90% |
|  | 6 400 | 20 | 2 | 90% |

**Table S2.** Results of paired t-tests comparing host growth before and after day 30 (all treatments combined).

| **Hosts** | **Mean difference** | **p (mean difference ≠ 0)** |
| --- | --- | --- |
| ***A. franciscana* males** |  |  |
| Growth between days 1 & 30 | 2.1 | < 0.0001 |
| Growth between days 30 & 60 | 0.0 | 0.87 |
| ***A. franciscana* females** |  |  |
| Growth between days 1 & 30 | 2.8 | < 0.0001 |
| Growth between days 30 & 60 | 0.3 | < 0.0001 |
| ***A. parthenogenetica*** |  |  |
| Growth between days 1 & 30 | 1.7 | < 0.0001 |
| Growth between days 30 & 60 | 0.3 | < 0.0001 |

**Table S3.** Model comparison: link between survival and infection success. For each host-parasite combination, these models grouped all the individuals exposed to that parasite (possible outcomes: uninfected or infected) and the control individuals for that host into the factor *Resistant-Infected-Control* (abbreviated Res-Inf-Ctrl). *Resistant-Infected-Control* was allowed to interact with all of the experimentally manipulated factors (the base model). The base model for *A. franciscana* included *Sex***Size class* and a frailty component for *Origin* (lognormal distribution, see Results). The base model for *A. parthenogenetica* included *Size class* and a frailty component for *Batch* (log-logistic distribution, see Results). We used contrast manipulation to detect how resistant, infected and control individuals differed (only models within ΔAICc = 3 of the best contrast-manipulated model are shown). Note that these analyses only included individuals that survived until at least day 15, when infection status could be definitively determined. *W* is the Akaike weight of each model.

| **Host-parasite combination** | **AICc** | **ΔAICc** | ***w*** |
| --- | --- | --- | --- |
| ***A. franciscana* exposed to *A. rigaudi*** |  |  |  |
| Base model + Res-Inf-Ctrl + Res-Inf-Ctrl : Sex  *Contrast manipulation: Ctrl* > *Inf* > *Res* | 1367.1  *1367.1* | 0 | 0.53 |
| Base model + Res-Inf-Ctrl | 1368.8 | 1.7 | 0.23 |
| Base model + Res-Inf-Ctrl + Res-Inf-Ctrl : Sex + Res-Inf-Ctrl : Size class | 1369.3 | 2.2 | 0.18 |
| Base model + Res-Inf-Ctrl + Res-Inf-Ctrl : Size class | 1371.5 | 4.4 | 0.06 |
| Base model | 1429.3 | 62.2 | 0.00 |
| ***A. parthenogenetica* exposed to *E. artemiae* - low spore dose** |  |  |  |
| Base model + Res-Inf-Ctrl  *Contrast manipulation: Inf* > *Ctrl* = *Res*  *Inf* > *Ctrl* > *Res*  *Inf* = *Ctrl* > *Res* | 641.5  *640.5*  *641.5*  *642.3* | 0 | 0.64 |
| Base model | 643.1 | 1.6 | 0.29 |
| Base model + Res-Inf-Ctrl + Res-Inf-Ctrl : Size class | 646.0 | 4.5 | 0.07 |

**Table S4 (next page).** Model comparison: link between reproduction and infection success. For each host-parasite combination, these models grouped all the individuals exposed to that parasite (possible outcomes: uninfected or infected) and the control individuals for that host into the factor *Resistant-Infected-Control* (abbreviated Res-Inf-Ctrl). *Resistant-Infected-Control* was allowed to interact with all of the experimentally manipulated factors (the base model). The base model for *A. franciscana* included *Size class* and *Origin* as a random or frailty component; the base model for *A. parthenogenetica* included *Size class* and *Batch* as a random effect. We used contrast manipulation to detect how resistant, infected and control individuals differed (only models within ΔAICc = 3 of the best contrast-manipulated model are shown). Note that these analyses only included individuals that survived until at least day 15, when infection status could be definitively determined. *w* is the Akaike weight of each model.

| **Host-parasite combination** | **AICc** | **ΔAICc** | ***w*** |
| --- | --- | --- | --- |
| ***A. franciscana* exposed to *A. rigaudi*: time until sexual maturity** |  |  |  |
| Base model + Res-Inf-Ctrl  *Contrast manipulation: Inf* = *Res* > *Ctrl*  *Inf* > *Res* > *Ctrl*  *Inf* > *Res* = *Inf* | 951.6  *949.7*  *951.8*  *953.2* | 0 | 0.82 |
| Base model + Res-Inf-Ctrl + Res-Inf-Ctrl : Size class | 954.6 | 3.0 | 0.18 |
| Base model | 974.4 | 22.8 | 0.00 |
| ***A. franciscana* exposed to *A. rigaudi*: probability of reproduction†** |  |  |  |
| Base model + Res-Inf-Ctrl  *Contrast manipulation: Ctrl* > *Inf* = *Res*  *Ctrl* > *Inf* > *Res* | 205.5  *204.0*  *205.5* | 0 | 0.75 |
| Base model + Res-Inf-Ctrl + Res-Inf-Ctrl : Size class | 207.8 | 2.3 | 0.24 |
| Base model | 239.9 | 34.4 | 0.00 |
| ***A. franciscana* exposed to *A. rigaudi*: clutch type** (higher = more nauplii)**†** |  |  |  |
| Base model + Res-Inf-Ctrl  *Contrast manipulation: Res* > *Inf* > *Ctrl*  *Res* = *Inf* > *Ctrl* | 306.7  *306.7*  *307.3* | 0 | 1.00 |
| Base model | 326.4 | 19.7 | 0.00 |
| ***A. franciscana* exposed to *A. rigaudi*: rate of offspring production*†** |  |  |  |
| Base model + Res-Inf-Ctrl  *Contrast manipulation: Res* > *Ctrl* = *Inf*  *Res* > *Ctrl* > *Inf*  *Res* *=* *Ctrl* *>* *Inf* | 96.0  *95.4*  *96.0*  *98.2* | 0 | 0.66 |
| Base model | 98.3 | 2.3 | 0.21 |
| Base model + Res-Inf-Ctrl + Res-Inf-Ctrl : Size class | 99.3 | 3.3 | 0.13 |
| ***A. franciscana* exposed to *A. rigaudi*: Fitness (LRS)*†** |  |  |  |
| Base model + Res-Inf-Ctrl  *Contrast manipulation: Ctrl* > *Res* = *Inf*  *Ctrl* > *Res* > *Inf* | 1097.6  *1095.5*  *1097.6* | 0 | 1.00 |
| Base model | 1145.7 | 51.7 | 0.00 |
| Base model + Res-Inf-Ctrl + Res-Inf-Ctrl : Size class | - | - | - |
| ***A. parthenogenetica* exposed to *E. artemiae* - low spore dose: clutch type** (higher = more nauplii) | | | |
| Base model + Res-Inf-Ctrl+ Res-Inf-Ctrl : Size class  *Contrast manipulation: order of effects dependent on Size class*  *order of effects dependent on Size class*  *order of effects dependent on Size class* | 315.6  *313.3*  *313.4*  *315.6* | 0 | 0.76 |
| Base model + Res-Inf-Ctrl | 318.3 | 2.7 | 0.20 |
| Base model | 321.2 | 5.6 | 0.05 |
| ***A. parthenogenetica* exposed to *E. artemiae* - low spore dose: rate of offspring production*** | | | |
| Base model | 181.1 | 0 | 0.96 |
| Base model + Res-Inf-Ctrl | 187.3 | 6.2 | 0.04 |
| Base model + Res-Inf-Ctrl + Res-Inf-Ctrl : Size class | 196.9 | 15.8 | 0.00 |
| ***A. parthenogenetica* exposed to *E. artemiae* - low spore dose: Fitness (LRS)*** | | | |
| Base model + Res-Inf-Ctrl  *Contrast manipulation: Inf* > *Res* = *Ctrl*  *Inf* = *Res* > *Ctrl*  *Inf* > *Res* > *Ctrl* | 1443.2  *1441.4*  *1441.8*  *1443.2* | 0 | 0.86 |
| Base model + Res-Inf-Ctrl + Res-Inf-Ctrl : Size class | 1446.8 | 3.6 | 0.14 |
| Base model | 1466.5 | 23.3 | 0.00 |

*Shown for the models that weighted nauplii and cysts equally; giving either offspring type a double weight produces qualitatively equivalent results. †Only two resistant females reproduced, so these results should be interpreted with caution.

**Figure S1. Spore production and host-to-host transmission success in the four host-parasite combinations.** These graphs relate the infection success (percentage of recipients infected) to the spore count in the corresponding spore sample (*ln* + 1 scale) for *A. rigaudi* (top) and *E. artemiae* (bottom). Note that the graphs are divided by recipient species, not donor species (see Methods). Each point represents a recipient group; overlapping points shade to black. Lines represent 2^nd^-degree polynomial local regression (LOESS) fittings.


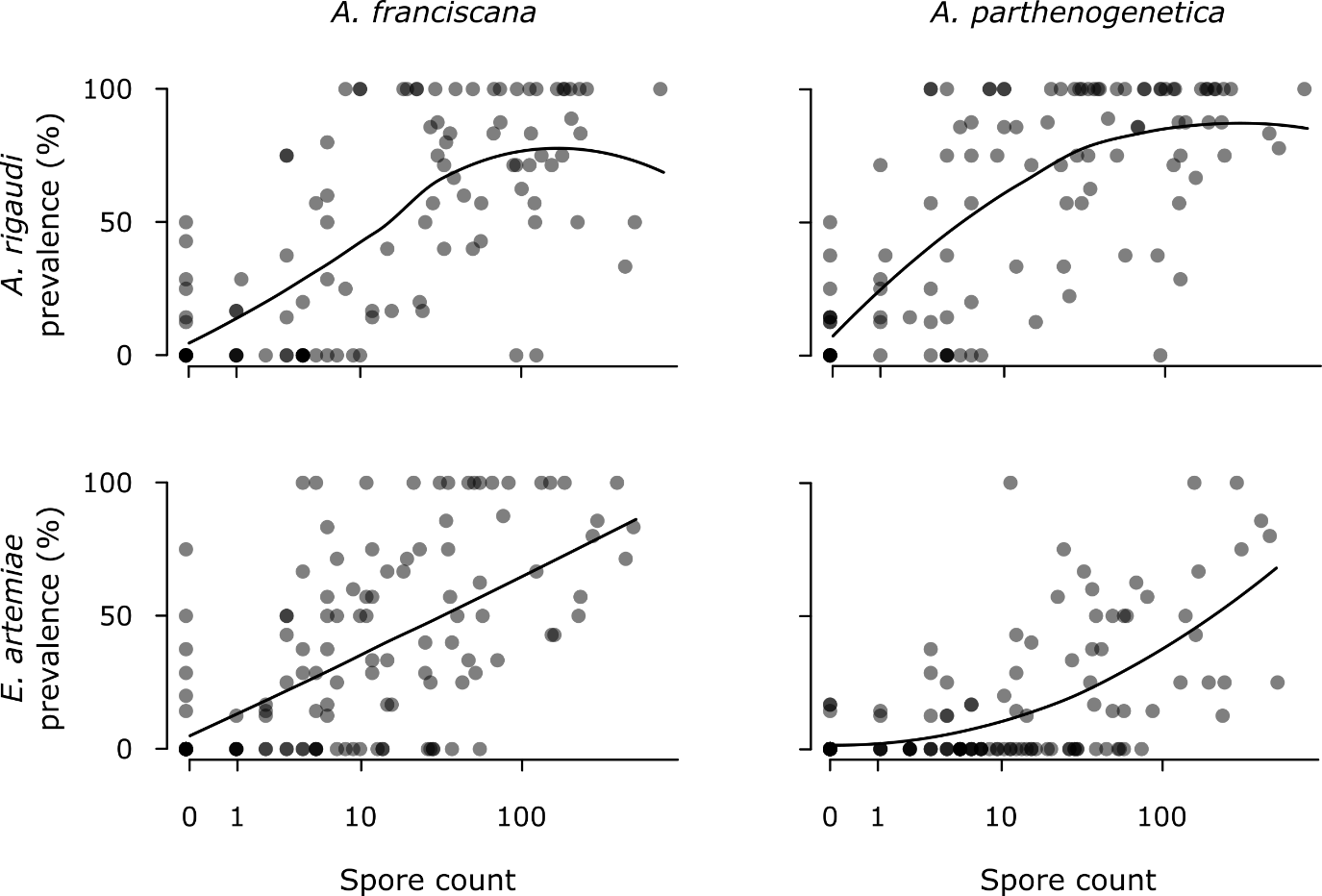


**Figure S2. Overall fitness measures of *A. rigaudi* (top) and *E. artemiae* (bottom) infections.** The asymptotic growth rate (*ln* + 1 scale) is shown as a function of the lifetime transmission success (*ln* + 1 scale). The asymptotic growth rate should be maximized during epidemics, while the lifetime transmission success, as an estimator of R_0_, should be maximized in endemic conditions. The median, first and third quartiles are shown by boxplots on the axes. For *A. parthenogenetica* infected with *E. artemiae*, the open circles and boxplot represent the females exposed to a high spore dose. Each point represents an infected host; overlapping points shade to black.


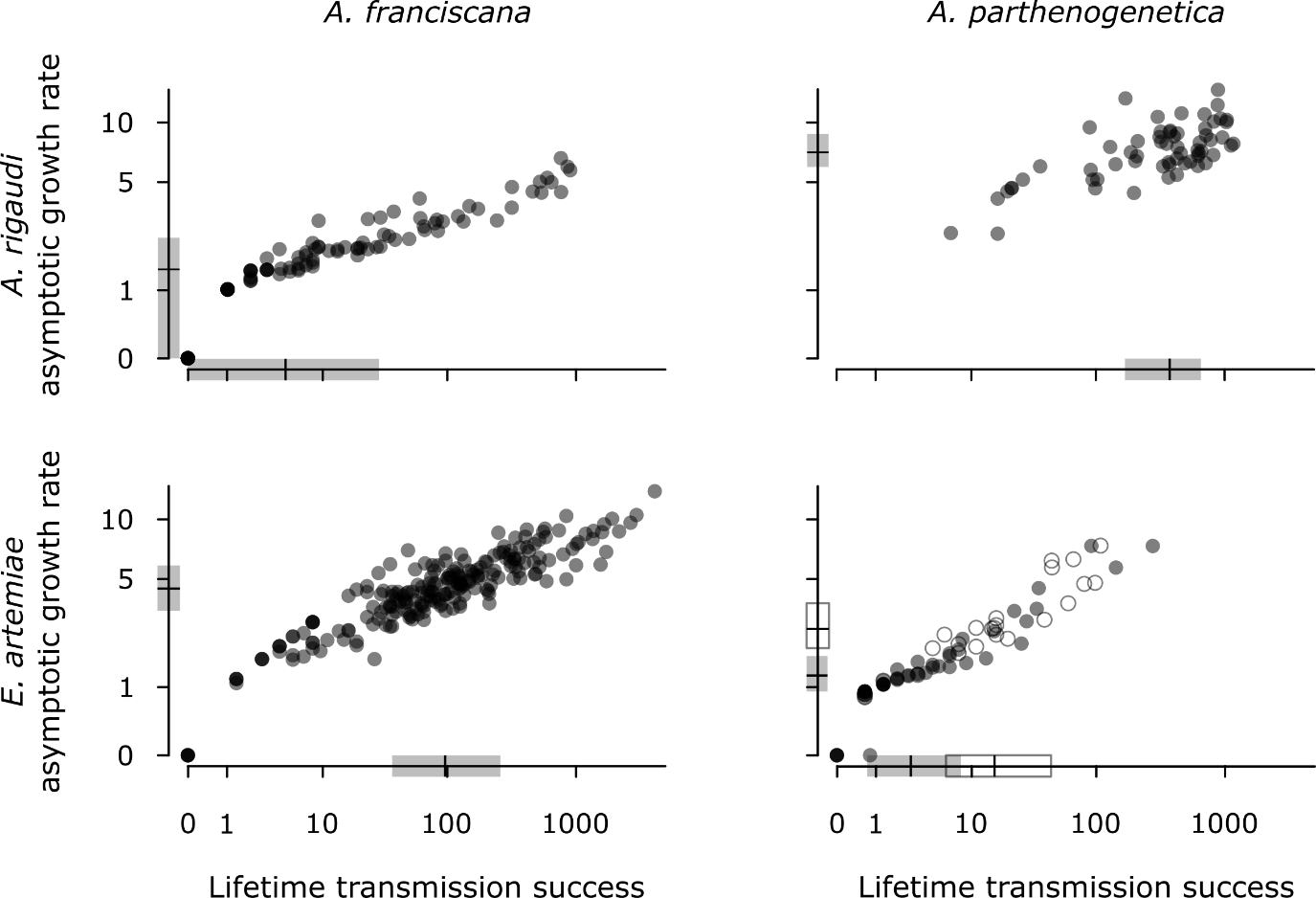


## Supplementary methods

We performed two experiments to investigate the life history and virulence of the microsporidians *A. rigaudi* and *E. artemiae* in their *Artemia* hosts. First, we used dose-response tests to quantify infectivity in each host-parasite combination. Second, we did a large-scale experimental infection experiment, tracking individual host growth, mortality, and reproduction, as well as parasite transmission, over a period of two months.

### Experimental conditions

The *Artemia* used in both experiments were raised in the lab in parasite-free conditions. *A. franciscana* were hatched from dormant cysts sampled from the saltern of Aigues-Mortes, France, and stored in dry conditions at 4 °C. We used three batches of cysts, sampled at the sites Caitive Nord or Caitive Sud in October 2013 or 2014. Cysts were hatched following the protocol described by Lievens et al. (2016). *A. parthenogenetica* were collected as live larvae from a mix of clones. The *A. parthenogenetica* clones were started by females collected in Aigues-Mortes, who were allowed to multiply and produce cysts in the lab; those cysts were then hatched to produce parasite-free stock lines. All *Artemia* were maintained at 23 ± 1 °C, in a parasite-free 90 ppt saline medium produced by diluting concentrated, autoclaved brine (Camargue Pêche, France) with deionized water. *Artemia* were fed *ad libitum* with freeze-dried microalgae (*Tetraselmis chuii*, Fitoplankton marino, Spain) dissolved in deionized water. Experimental conditions matched the cultivation conditions, except that feeding was regulated (see below).

We created stocks of *A. rigaudi* and *E. artemiae* for use in the experiments by combining infected *Artemia* from various sites in Aigues-Mortes between October 2014 and March 2015. We added new infected hosts to the stocks whenever we found field populations that were heavily infected with either *A. rigaudi* or *E. artemiae*. We also regularly added uninfected, lab-bred *Artemia* to help maintain the infection. We selected both infected *A. franciscana* and infected *A. parthenogenetica* from the field, and maintained each stock population on a mix of *A. franciscana* and *A. parthenogenetica* hosts (n_hosts_ at any given time = ~20-~50 per microsporidian species). Thus, our stocks contained a mix of spores from different field sites and times, collected from and propagated on both host species.

### Spore collection and quantification

To produce the inocula for Experiments 1 and 2, we collected spores from the lab stocks of *A. rigaudi* and *E. artemiae* described above. The stock hosts were kept in large separating funnels, so that their feces (containing spores) settled down into the funnel’s tube and could be collected easily. For our experiments, we collected feces produced over 20-hour periods (feces suspended in ~15 mL). Because fecal aggregates can trap spores and skew concentration estimates, we homogenized the fecal solutions by dividing them into 1.2 mL Qiagen Collection Microtubes, adding two 4 mm stainless steel beads to each tube, and shaking them at 30 Hz for 30 s. Once homogenized, the fecal solutions were recombined to their original volume. To quantify the spore concentration in the fecal solutions, we took 1 mL subsamples and added 10 µL 1X Calcofluor White Stain (18909 Sigma-Aldrich, USA) to each. After staining for 10 min, we rinsed the subsamples by centrifuging them for 8 min at 10 000 g, replacing 910 µL of the supernate with 900 µL deionized water and vortexing well. We then concentrated the subsamples to 20X by repeating the centrifugation step and removing 950 µL of the supernate. Finally, we estimated the concentration by counting the number of spores in 0.1 µL on a Quick Read counting slide (Dominique Dutscher) under a Zeiss AX10 fluorescence microscope (10x 40x magnification; excitation at 365 nm; Zeiss filter set: 62 HE BFP + GFP + HcRed shift free (E)). We repeated the counts twice (for Experiment 2) or thrice (for Experiment 1); the spore concentration per µL in the unconcentrated fecal solutions was then equal to (mean of the spore counts*10)/20. Finally, we added 90 ppt clean saline medium to the homogenized fecal solutions until the correct concentration for inoculation was reached.

### Experiment 1: Infectivity

#### Experimental design and execution

Previously, we studied the infectivity of *A. rigaudi* and *E. artemiae* using single, uncontrolled spore doses (Rode *et al.* 2013). Here, we quantified infectivity more precisely by exposing individual *A. parthenogenetica* and *A. franciscana* to a range of controlled spore doses and measuring the proportion of infected individuals.

We exposed experimental hosts to doses of 0, 400, 800, 1 600, 3 200 and 6 400 spores per individual. To ensure hosts ingested all spores, each host was first exposed in a highly-concentrated medium: individuals were placed in 2 mL Eppendorf tubes with 0.45 mL spore solution, 1 mL extra brine and 0.25 mL algal solution (3.4*10^9^ *T. chuii* cells/L deionized water). After two days, hosts were transferred to 40 mL glasses containing 20 mL brine and the infection was allowed to incubate for three more days; hosts were fed a total of 1 mL algal solution over the three days. Surviving hosts were then sacrificed and tested for the presence of *A. rigaudi* or *E. artemiae* by PCR (following Rode *et al.* 2013). Treatments were replicated 20 times, except when spore availability was limiting (*E. rigaudi* on *A. parthenogenetica*: 16, 8 and 4 replicates for the doses 400, 3 200 and 6 400 spores per individual, respectively). All hosts were ~4 weeks old and measured between 5 and 8 mm; *A. franciscana* hosts were mixed males and females.

#### Statistical analyses

To analyze the dose-response curves, we used four-parameter log-logistic modeling in R (package drc, Ritz & Strebig 2005; R Core Team 2014). In these models, the four parameters determining the shape of the sigmoidal curve are: the lower limit (set to 0 in our case), the upper limit, the slope around the point of inflection, and the point of inflection (which here is the same as the ED_50_). The (binomial) response variable was the number of individuals that were infected vs. uninfected. Because we did not perform the *A. parthenogenetica* and *A. franciscana* experiments at the same time, we could not control for environmental effects. Thus, we simply tested if the dose-response curves for *A. rigaudi* and *E. artemiae* were different within each host species. To do this, we fit models that did or did not include a ‘microsporidian species’ effect and compared the two using a likelihood ratio test. If the effect was significant, we went on to compare the parameters of the two resulting curves (‘compParm’ function in the drc package).

### Experiment 2: Virulence and transmission

#### Experimental design and execution

To quantify the virulence and transmission rates of *A. rigaudi* and *E. artemiae*, we experimentally infected individual *Artemia* with controlled spore doses. We then tracked their survival, growth, reproductive output, and spore production over a two-month period. We also quantified host-to-host transmission at two time points.

*A. franciscana* males, *A. franciscana* females, and *A. parthenogenetica* females were divided into three treatments: ‘Controls’, ‘Exposure to *A. rigaudi*, and ‘Exposure to *E. artemiae*’, which were replicated as permitted by spore and host availability (Table 1). *A. franciscana* hosts were subdivided into three blocks, determined by their origin: Caitive Nord 2013, Caitive Nord 2014, or Caitive Sud 2014. *A. parthenogenetica* hosts were subdivided into two blocks, determined by the age of their batch: 34 ± 2 or 26 ± 2 days (because the relative contribution of the different clones to the batches was not controlled, the genotype frequencies of these groups could differ). All hosts were subadults (adult body plan but sexually immature). All *A. franciscana* were aged 38 ± 1 days and measured 4.5 or 5.0 mm; *A. parthenogenetica* measured 6.5, 7.0 or 7.5 mm. Size classes were evenly distributed across blocks and treatments.

We exposed experimental hosts to spore doses designed to be comparable while maximizing infection rate (see results of Experiment 1): 3 000 spores/individual for *A. rigaudi* and 2 500 spores/individual for *E. artemiae*. Because *A. parthenogenetica* had low infection rates with *E. artemiae*, a separate set of *A. parthenogenetica* was infected with 10 000 *E. artemiae* spores per individual (Table 1). To ensure hosts ingested all spores, each host was exposed in a highly-concentrated medium over a two-day period: individuals were placed in 2 mL Eppendorf tubes with 0.37 mL spore solution and 1.25 mL brine containing 2.6*10^6^ *T. chuii* cells.

After exposure (= on day 1 of the experiment), individuals were transferred to open tubes, which rested upright in 40 mL plastic cups containing 20 mL of brine. The lower end of the tube was fitted with a 1x1 mm net. The netting prevented experimental (adult) individuals from swimming to the bottom of the cup, while allowing spores, feces, and offspring to pass through; this limited secondary infections from a host’s own feces. Cups were randomly placed in trays, which were routinely rotated to standardize effects of room placement. Water was changed every five days. Hosts were fed 0.5 mL algal solution daily (2.6*10^9^ *T. chuii* cells/L deionized water); this feeding regime corresponds to half of the maximum ingestion rate of an adult *Artemia* (Reeve 1963) and has been shown to reveal energetic trade-offs (Rode *et al.* 2011). We ended our experiment after 60 days, at which point surviving individuals were sacrificed and tested for infection by PCR (following Rode *et al.* 2013).

To quantify the effects of infection on the hosts, we tracked the growth, survival, and reproduction of the experimental individuals. Body length was recorded on days 30 and 60. Survival was recorded daily; dead individuals were tested for infection by PCR (following Rode *et al.* 2013). We did not track reproduction for males, because male reproductive success is heavily influenced by the female partner (e.g. female clutch size). For females, measures of reproductive success were recorded daily, including date of sexual maturity (first detection of a fully-formed ovisac or of yolk accumulation in oocytes, Metalli & Ballardin 1970), clutch date, clutch type, and clutch size. *Artemia* females are iteroparous, producing on average one clutch per five days (Bowen 1962; Metalli & Ballardin 1970). Clutches may be of two types: live larvae (‘nauplii’), or dormant encysted embryos (‘cysts’). Neonatal nauplii are barely visible to the eye and have high death rates. For ease of measurement, therefore, clutches of nauplii were counted five days after sighting. During these five days nauplii were in competition for resources with their mother (plus an additional male if *A. franciscana*, see below). However, mothers were removed at each water change, which could happen before the clutch had reached the five-day mark. In these cases, we placed a new tube containing one (or two, if *A. franciscana*) adult male *Artemia* above the nauplii to ensure the same level of food competition. Most of these substitute males were uninfected. When obliged by logistical reasons to use males that were infected with *A. rigaudi* or *E. artemiae*, we made sure only to place them with nauplii from respectively *A. rigaudi*- and *E. artemiae*-exposed mothers. The 5-day survival of these nauplii was therefore unbiased by exposure to ‘foreign’ parasites.

While *A. parthenogenetica* females reproduce in isolation, *A. franciscana* females need to be fertilized before each clutch (Bowen 1962). We therefore added mature *A. franciscana* males from parasite-free lab stocks to each tube containing an *A. franciscana* female. To prevent cross-contamination between the male and the female, exposed males were removed and new uninfected males added every five days (five-day estimate based on infection detection time as found by Rode *et al.* 2013). Male *Artemia* mate-guard by clasping females around the abdomen (Bowen 1962), and forcible removal may be harmful to both partners. To avoid this, males found mate-guarding on the fifth day were given up to two extra days with the female, after which they were forcibly removed. Couples were fed twice the individual food allocation.

To estimate parasite fitness, we estimated spore production at regular points throughout the experiment. To do this, we collected 1 mL of feces (containing parasite spores) from every experimentally infected host at every water change. Samples were stored in 1.2 mL Qiagen Collection Microtubes and refrigerated until the spore concentration could be quantified. To measure the spore concentration, we homogenized and stained each sample as described above, with minor differences in the centrifugation steps (16 min at 5 000 g) and the final concentration (concentrated to 14.3X by removing 930 µL of the supernate). Spores were counted once per sample, as described above. Because counting spores is labor-intensive, we restricted our efforts to the feces samples collected on days 15, 30, 45 and 60.

We also investigated the host-to-host transmission success of the parasites and its relation to spore production. On days 30 and 60, we allowed a subset of experimental hosts (hereafter the ‘donors’) to infect groups of uninfected ‘recipient’ hosts for 24 hours. Donors were first placed with either eight *A. franciscana* or eight *A. parthenogenetica* recipients; after 24 hours, the donor was removed and placed with a new group of recipients of the other species. All recipient hosts were taken at random from uninfected lab stocks of varying ages and sizes (min = 4 mm, max = 10 mm). The donor host was separated from the recipients by a 1x1 mm net; recipients swam underneath them in 40 mL of brine. Infection was allowed to incubate in the recipients for six days after the donor was removed; surviving recipients were then sacrificed and PCR-tested for infection (following Rode *et al.* 2013). The prevalence of infection in recipient individuals could then be compared to the number of spores counted in the feces samples on day 30 or 60.

A key aspect of infection follow-up experiments is knowing which individuals were infected after exposure to the parasite, and which were not. In our experiment, testing by PCR was often not sufficient to determine if an individual was infected, because individuals that died before day 60 often had quickly decaying corpses and thus degraded DNA. We therefore considered that an individual was infected if it tested positive by PCR or produced spores or transmitted the infection to a recipient host. If none of these requirements were met, we considered that the individual was not infected. By applying these criteria, we could be sure of the infection status for almost all individuals that died on or after day 15 (the first spore collection date); for any individuals who died before day 15 and who tested negative by PCR, we could not exclude the possibility that they were infected.

#### Statistical analyses: virulence & transmission (= detailed description of analyses in Table 2)

We analyzed the results of this experiment in two major parts. First, we examined the virulence of infections (effect of the parasite on host survival, growth, reproduction, and overall fitness). Second, we analyzed parasite transmission (spore production rate, infectiousness, and overall fitness). Analyses were run in R version 3.4.2 (R Core Team 2014) using the packages lme4 (linear mixed models, Bates *et al.* 2015), survival (survival analyses, Therneau 2014), pscl (hurdle models, Zeileis *et al.* 2008), and multcomp (fuction “glht” for post-hoc testing, Hothorn *et al.* 2008).

First, we analyzed the virulence of infections (effect of the parasite on host survival, growth, reproduction, and overall fitness). This was done separately for *A. franciscana* and *A. parthenogenetica*. *A. parthenogenetica* exposed to low and high doses of *E. artemiae* were treated as separate treatments. Unless otherwise specified, our analyses proceeded as follows: we included our experimentally manipulated factors in a full regression model, used likelihood ratio tests to test their significance, and where relevant carried out post-hoc comparisons using Dunnett’s comparisons with a control (i.e. infected-with-*A. rigaudi* vs. controls, infected-with-*E. artemiae* vs. controls). Importantly, we only analyzed virulence once we could be certain of individuals’ infection status. To do this, we excluded all individuals that died before day 15 (see Methods > Experiment 2: Virulence and transmission > Experimental design and execution), and only compared infected with control individuals. To make sure that we were not missing important events occurring before this cutoff, we repeated all statistical models for exposed vs. control individuals that died before day 15.

We analyzed host survival using parametric survival models. We established a full fixed-effects model for each host species, then determined the best-fitting parametric distribution (Weibull, exponential, extreme, Gaussian, logistic, lognormal, log-logistic, Rayleigh) using the corrected AIC (Hurvich & Tsai 1989). We then tested the significance of the predictive effects as described above. Finally, we confirmed the fit of the model by performing a goodness-of-fit test (comparing the likelihood of the observed data with the likelihood distribution of simulated datasets based on the model predictions). The full model for *A. franciscana* included *Treatment*, *Sex*, *Size class*, and all double interactions. The full model for *A. parthenogenetica* included *Treatment*, *Size class*, and their interaction. *Origin* and *Batch* were included as frailty components for *A. franciscana* and *A. parthenogenetica*, respectively, as they could introduce heterogeneity in mortality rates. Data were right-censored on day 60.

To test the effects of parasite infection on growth, we first checked whether there was significant growth between days 1 & 30 and days 30 & 60 (paired t-tests of the size difference between day 30 & 1 and day 60 & 30). Most growth occurred during the first month (see Results), so we analyzed growth between days 1 & 30 further using linear mixed models. For *A. franciscana*, we looked at the effects of the fixed effects *Sex*, *Treatment*, *Size class* and all their interactions, with *Origin* as a random effect. For *A. parthenogenetica*, the full model included *Treatment*, *Size class* and their interaction as fixed effects, and *Batch* as a random effect.

To analyze (female) reproductive success, we decomposed female reproduction into a) time until sexual maturity, b) the probability of producing a clutch, c) the rate of offspring production, d) the timing of offspring production, and e) the type of offspring produced. All models included *Treatment*, *Size class*, and their interaction as fixed effects, and *Origin* or *Batch* as random (or frailty) effects. The response variables and statistical models were as follows. a) Time until sexual maturity: the number of days until females became sexually mature, analyzed using parametric survival models. As above, we first determined the best survival distributions to use, then tested the significance of the predictive effects. Females were right-censored in case of death. b) Probability of producing a clutch: a binary variable describing whether a female produced a clutch during the experiment or not, analyzed using generalized linear mixed models with a Bernouilli distribution. c) Rate of offspring production: for females that produced at least one clutch, the total number of offspring divided by the length of the reproductive period. The length of the reproductive period was defined as the difference between the date of death (or censoring) and the date of maturity. The data were analyzed using linear mixed models. Offspring could be nauplii or cysts, and these two offspring types were not directly comparable (they probably require different amounts of energy to produce, and we allowed mortality to occur before counting nauplii). To account for this, we repeated the analyses with nauplii weighted twice, equally, or half as much as cysts, and based our conclusions on the overall pattern. d) Timing of offspring production: for females that produced at least one clutch, the clutch size through time. Clutch size was modelled as a quadratic function of clutch date, with clutch date expressed as the elapsed proportion of the female’s reproductive period (e.g. for two females reproducing on the 10^th^ day of sexual maturity, where one died on the 20^th^ day and one was censored on the 40^th^, the elapsed proportions would be 0.5 and 0.25). Timing was analyzed using generalized linear mixed models with a negative binomial distribution; *Individual* was included as a random variable to control for pseudoreplication. As in (c), we ran models where nauplii were weighted twice, equally, or half as much as cysts, and based our conclusions on the overall pattern. e) Type of offspring produced: for females that produced at least one clutch, a binomial combination of the number of clutches consisting of nauplii vs. cysts, analyzed using generalized linear mixed models.

As a final virulence measure, we estimated the fitness of (female) hosts. Our fitness proxy was the lifetime reproductive success (LRS), calculated as the total number of offspring produced over the study period. This produced a zero-inflated count distribution, to which we fit negative binomial hurdle models. The full models included *Treatment*, *Size class*, and their interaction as fixed effects; random effects (such as *Origin* and *Batch*) were not supported by the package. As above, we ran models where nauplii were weighted twice, equally, and half as much as cysts, and based our conclusions on the overall pattern.

Next, we analyzed the parasites’ transmission (spore production rate, infectiousness of a single spore, and overall fitness). These analyses were combined for infections in *A. franciscana* and *A. parthenogenetica*. Unless otherwise specified, we included our experimentally manipulated factors in a full regression model, and used likelihood ratio tests to test their significance. If relevant, post-hoc comparisons were carried out using Tukey comparisons.

To estimate the infectiousness of a single spore, we used the results of the transmission assay. We assumed that the establishment of microsporidian infections follows an independent-action model with birth-death processes. This model assumes that a parasite population grows in the host until it reaches an infective threshold, at which point the infection is considered to be established (Schmid-Hempel 2011). In our assay, we considered that an infection was established when we could detect it; in other words, the infective threshold corresponded to the threshold for PCR detection (estimated at ~1 000 spores inside the host’s body, unpublished data). In these models, the probability per spore to start an infection, *p*, is equal to $-\ln\left( \frac{noninfected recipients}{total recipients} \right)/D$ where *D* is the spore dose (Schmid-Hempel 2011). In our transmission assay, *D* can be approximated by the number of spores in the fecal sample taken from the donor at the start of the assay (= spore count transformed to spores/mL, or * 700), divided by 5*8 = 40 (fecal samples accumulated over a 5-day period but we only exposed recipients for one day; the inoculum was shared amongst 8 recipients). We calculated a value of *p* for every replicate in the transmission assay; *p* was then analyzed using linear mixed models. The model included *Recipient species*, *Parasite species*, and their interaction as fixed effects; an *Individual*-level random effect was included to control for pseudoreplication (each donor host was used to infect a group of *A. franciscana* and a group of *A. parthenogenetica* recipients; some donors were also re-used in the transmission assays on day 30 and 60).

We then tested whether the rate of spore production was dependent on the host-parasite combination. We used *Spore count*, the number of spores counted in the fecal sample, as the response variable in a generalized linear mixed model with a negative binomial distribution. We did not transform the spore count to spores/mL (≈ spore count * 700) to avoid skewing the error distribution. The fixed effects were *Host species*, *Parasite species*, and their interaction; an *Individual*-level random effect was included to control for pseudoreplication. To avoid comparing apples with oranges, we excluded *A. parthenogenetica* that had been exposed to 10 000 *E. artemiae* spores from this model. However, we tested separately whether the rate of spore production differed for *A. parthenogenetica* infected with different doses of *E. artemiae* (equivalent model with fixed effect *Dose*). Spore production analyses were carried out for infected hosts only.

As a final measure of parasite success, we investigated parasite fitness in the different host-parasite combinations. For each established infection (i.e. each infected host), we used two measures of spore production as proxies for parasite fitness. First, we calculated a proxy for the ‘lifetime transmission success’: we summed the number of spores in the fecal samples taken on days 15, 30, 45 and 60 for each infection, then corrected this cumulative spore count by *p*, the average infectiousness of a single spore in a given host-parasite combination (as calculated above). Second, we calculated an asymptotic growth rate by computing the dominant eigenvalue of a standard Leslie matrix,

$\left[ \begin{matrix} \begin{matrix} 0 & n_{15}*p & n_{30}*p & n_{45}*p & n_{60}*p \\ s_{15} & 0 & 0 & 0 & 0 \\ 0 & s_{30} & 0 & 0 & 0 \\ 0 & 0 & s_{45} & 0 & 0 \\ 0 & 0 & 0 & s_{60} & 0 \end{matrix} \end{matrix} \right]$,

where *n_i_* is the number of spores in the fecal sample on day *i*, *p* is the average infectiousness of a single spore in that host-parasite combination (as calculated above), and *s_i_* describes whether the host survived until day *i* (1) or not (0). Both proxies implicitly account for host density and spore encounter rate, as *p* is calculated for the specific density of the transmission assay. While the lifetime transmission success is a measure of the basic reproduction number R_0_, which describes parasite fitness under stable endemic conditions, the asymptotic growth rate is a measure of the net population growth rate, which describes fitness under epidemic conditions (Frank 1996; Hethcote 2000); we included both measures because either situation can occur in the field. It should be noted that we calculate these proxies to compare parasite fitness under these specific standardized conditions, not to serve as estimates of parasite fitness in the field. Parasite fitness in the field may differ due to e.g. the release of spores from dead hosts, spore sedimentation and spore death, variation in host demography (changes in age structure and density across seasons and basins) and variation in host ‘quality’ (presence of other parasites, heterogeneous nutrition levels, different development time at different temperatures, etc.) (Alizon & Michalakis 2015). We compared the two measures across host-parasite combinations using non-parametric Kruskal-Wallis tests with Dunn’s post hoc testing (R package PMCMR, Pohlert 2014). *A. parthenogenetica* exposed to low and high spore doses of *E. artemiae* were treated separately.

#### Statistical analyses: infection vs. resistance

In most of the experimental host-parasite combinations, a subset of exposed hosts did not become (detectably) infected. Hereafter, we refer to these individuals as resistant, because we found *a posteriori* differences in the proportion of such individuals across host-parasite combinations, and in their life history traits compared to infected individuals and controls. As above, the analyses of these two aspects excluded all individuals who died before infection status could be definitively determined, i.e. those that died before day 15 of the experiment.

We analyzed the distribution of resistance across host-parasite combinations. Within each host species, we used χ^2^ tests to compare the numbers of resistant and infected hosts after exposure to *A. rigaudi* and *E. artemiae*. We also used χ^2^ tests to test for an effect of sex on the probability of resistance to each parasite in *A. franciscana*, and for an effect of spore dose on the probability of resistance to *E. artemiae* in *A. parthenogenetica*.

There was substantial variation in infection outcome for the combinations *A. franciscana*-*A. rigaudi*, and *A. parthenogenetica*-*E. artemiae* (low dose) (see Results). Because costs of resistance are a common aspect of host-parasite interactions (Schmid-Hempel 2003), we investigated whether resistance was related to host fitness in these combinations. To do this, we repeated the survival and reproduction analyses described above, with an added *Resistant-Infected-Control* factor. We added or excluded this factor and its interactions with the other fixed effects, then compared all models using the corrected AIC. In this way, we investigated whether the outcome of infection explained a significant part of the variation in host traits after the experimentally manipulated factors were taken into account. If the *Resistant-Infected-Control* factor was maintained in the best models, we used contrast manipulation and AICc-based model comparison to detect how the three host categories (*Resistant*, *Infected*, *Control*) differed.

## Supplementary references

Alizon, S. & Michalakis, Y. (2015). Adaptive virulence evolution: the good old fitness-based approach. *Trends Ecol. Evol.*, 30, 248–254.

Bates, D., Maechler, M., Bolker, B. & Walker, S. (2015). Fitting Linear Mixed-Effects Models Using lme4. *J. Stat. Softw.*, 67, 1–48.

Bowen, S.T. (1962). The genetics of Artemia salina. I. The reproductive cycle. *Biol. Bull.*, 122, 25–32.

Frank, S.A. (1996). Models of parasite virulence. *Q. Rev. Biol.*, 71, 37–78.

Hethcote, H.W. (2000). The Mathematics of Infectious Diseases. *Soc. Ind. Appl. Math. Rev.*, 42, 599–653.

Hothorn, T., Bretz, F. & Westfall, P. (2008). Simultaneous Inference in General Parametric Models. *Biometrical J.*, 50, 346–363.

Hurvich, C.M. & Tsai, C.L. (1989). Regression and Time-Series Model Selection in Small Samples. *Biometrika*, 76, 297–307.

Lievens, E.J.P., Henriques, G.J.B., Michalakis, Y. & Lenormand, T. (2016). Maladaptive sex ratio adjustment in the invasive brine shrimp Artemia franciscana. *Curr. Biol.*, 26, 1463–1467.

Metalli, P. & Ballardin, E. (1970). Radiobiology of Artemia: radiation effects and ploidy. *Curr. Top. Radiat. Res. Q.*, 7, 181–240.

Pohlert, T. (2014). The Pairwise Multiple Comparison of Mean Ranks Package (PMCMR).

R Core Team. (2014). R: A language and environment for statistical computing.

Reeve, M.R. (1963). The filter-feeding of Artemia I. In pure cultures of plant cells. *J. Exp. Biol.*, 40, 195–205.

Ritz, C. & Strebig, J.C. (2005). Bioassay Analysis using R. *J. Stat. Softw.*, 12.

Rode, N.O., Charmantier, A. & Lenormand, T. (2011). Male-female coevolution in the wild: evidence from a time series in Artemia franciscana. *Evolution*, 65, 2881–2892.

Rode, N.O., Landes, J., Lievens, E.J.P., Flaven, E., Segard, A., Jabbour-Zahab, R., *et al.* (2013). Cytological, molecular and life cycle characterization of Anostracospora rigaudi n. g., n. sp. and Enterocytospora artemiae n. g., n. sp., two new microsporidian parasites infecting gut tissues of the brine shrimp Artemia. *Parasitology*, 140, 1168–85.

Schmid-Hempel, P. (2003). Variation in immune defence as a question of evolutionary ecology. *Proc. R. Soc. London, Ser. B*, 270, 357–366.

Schmid-Hempel, P. (2011). *Evolutionary parasitology: the integrated study of infections, immunology, and genetics*. Oxford University Press, Oxford, UK.

Therneau, T.M. (2014). A Package for Survival Analysis in S.

Zeileis, A., Kleiber, C. & Jackman, S. (2008). Regression Models for Count Data in R. *J. Stat. Softw.*, 27, 1–25.
